# Heterologous (Over) Expression of Human SoLute Carrier (SLC) in Yeast: A Well-Recognized Tool for Human Transporter Function/Structure Studies

**DOI:** 10.3390/life12081206

**Published:** 2022-08-08

**Authors:** Lorena Pochini, Michele Galluccio

**Affiliations:** Laboratory of Biochemistry, Molecular Biotechnology, and Molecular Biology, Department of Biology, Ecology and Earth Sciences (DiBEST), University of Calabria, Via P. Bucci 4c, 87036 Rende, Italy

**Keywords:** yeast, expression, SLC transporter

## Abstract

For more than 20 years, yeast has been a widely used system for the expression of human membrane transporters. Among them, more than 400 are members of the largest transporter family, the SLC superfamily. SLCs play critical roles in maintaining cellular homeostasis by transporting nutrients, ions, and waste products. Based on their involvement in drug absorption and in several human diseases, they are considered emerging therapeutic targets. Despite their critical role in human health, a large part of SLCs’ is ‘orphans’ for substrate specificity or function. Moreover, very few data are available concerning their 3D structure. On the basis of the human health benefits of filling these knowledge gaps, an understanding of protein expression in systems that allow functional production of these proteins is essential. Among the 500 known yeast species, *S. cerevisiae* and *P. pastoris* represent those most employed for this purpose. This review aims to provide a comprehensive state-of-the-art on the attempts of human SLC expression performed by exploiting yeast. The collected data will hopefully be useful for guiding new attempts in SLCs expression with the aim to reveal new fundamental data that could lead to potential effects on human health.

## 1. Introduction

Transporters account for 10% of the human genome. They regulate the flux of different molecules such as sugars, amino acids, ions, lipids, vitamins, and catabolites through plasma and subcellular membranes [1]. In addition to their physiological roles, many transporters are involved in drug absorption, distribution, metabolism, and excretion (ADME) processes. Moreover, many drugs can interact with membrane transporters, leading to side effects [2]. Indeed, about 60% of FDA-approved drug targets are membrane proteins, including a growing number of transporters [3,4]. A well-established functional classification categorizes transport proteins in channels and permeases. Channels catalyze the transport of ions down their electrochemical gradient, with a turnover rate up to 10^8^ s^−1^. Permeases are involved in the transport of many different molecules with a lower turnover rate. On the basis of the transport driving force, permeases can be subdivided in primary and secondary active transporters [5]. The first use ATP hydrolysis to drive the transport of molecules against their concentration gradient. The secondary active transporters retrieve the driving force from the concentration gradient of the transported substrates and are grouped into symporters, antiporters, and uniporters. The uniporters catalyze the substrate movement in a thermodynamically favorable direction; the symporters or antiporters catalyze co-transport of more molecules in the same or in the opposite direction, respectively [5,6]. Among the transporters, the SoLute Carrier (SLC) superfamily currently includes 458 proteins grouped into 65 families [7,8]. Member proteins within each family share at least 20–25% sequence similarity with at least one other member of the family [7,9]. The number of members within the SLC families is heterogeneous, ranging from only one (SLC32, SLC40, SLC48, SLC50, SLC53, SLC61, SLC62, and SLC64) to 53 members for the largest family: SLC25, the mitochondrial carrier family [7]. The members of this superfamily act as secondary active transporters [10]. Regarding the molecular mechanism of transport, the secondary active transporters use the “alternating access” transport mechanism in which the ligand binding site is available on only one side of the membrane at a time; then, as a consequence of the substrate interaction, a protein conformational change does occur and the substrate is transported to the other side of the membrane [11]. Three major types of alternating access mechanisms have been described for SLC transporters: the rocker-switch, the gated pore and the elevator mechanism [10]. Analyzing hydropathy plots, the SLC transporters are predicted to present between 1–16 transmembrane domains (TMD), although in more than 80% of the members the number of TMD is between 7 and 12 [12]. Despite their role in human pathophysiology and the efforts employed in fulfilling knowledge of them, about 30% of the SLC members are still “orphans”, with no structural and functional data available [7,13]. The reason for this gap in information lies in the difficulty in expressing, purifying, and assaying the function of this kind of proteins [14]. These difficulties arise from the hydrophobic nature of the TMD and, in some cases, from very large (about 100 amino acids) hydrophilic moieties which connect TMDs [15,16]. Different expression hosts, either prokaryotic or eukaryotic, have been adopted with success allowing structure/function studies on SLC transporters such as, *E. coli* [17], *L. lactis* [18], *S. cerevisiae* [19], *P. pastoris* [20], *S. frugiperda* [21], HEK293F [22]. However, less than 10% of the human SLCs structure has been solved [23]. Due to very low cultivation costs, fast growth, easy handiness, and absence of toxicity, *Escherichia coli* is the most used host for protein over-expression. In particular, BL21(DE3) and its derivative strains are the most commonly used for heterologous membrane protein production [23]. Even though widely exploited for protein over-expression, in the case of human membrane transporters, *E. coli* could be ineffective, leading to the inclusion of a bodies formation from which it is difficult to recover the target protein in an active folded form [24,25]. On the other side, expression in insect or mammalian cell systems is characterized by high cultivation costs, slow growth, and needs special equipment, often resulting in low yield. A good middle ground is represented by the yeast expression system [26] which combines the advantages of ease of manipulation, fast growth, low cost of production, eukaryotic protein processing [27] (which can facilitate the expression of correctly-folded eukaryotic proteins) [28], heterodimer formation, and prosthetic group association to the recombinant proteins [29]. Moreover, the localization of an expressed membrane transporter in the yeast membrane can facilitate its recovery in a folded state [30]. Indeed, in this milieu, the human membrane proteins can find a more similar phospholipid/sterol composition with respect to the bacterial hosts [31]; furthermore, the possibility of increasing culture volume to obtain large quantities of folded protein [31,32] can be exploited either for functional or structural studies [20,33,34]. Indeed, yeast and especially *P. pastoris* can reach extremely high biomass, thus obviating the need to achieve extremely high levels of overexpression that could result in the aggregation and denaturation of membrane proteins.

The following sections will provide a comprehensive overview of the employment of yeast in human SLC transporters expression and the resulting most relevant knowledge acquired on their function.

## 2. Yeasts as a System for Heterologous Expression of Human SLC Transporters

**Figure 1:**
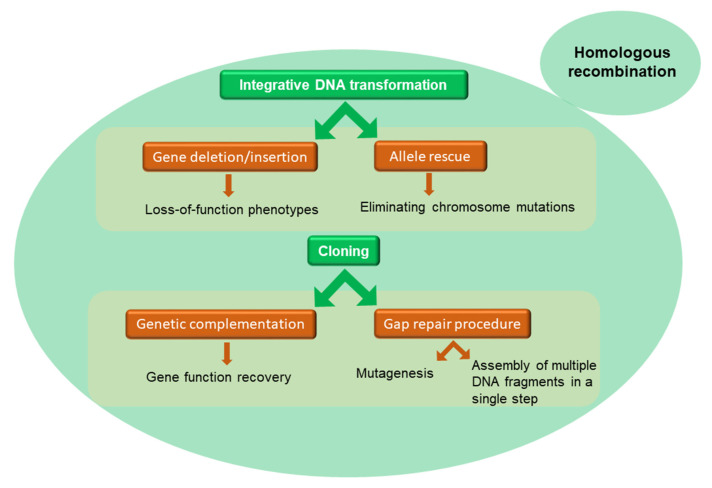
Uses of the homologous recombination in yeast.

A similar cellular architecture with respect to higher eukaryotes, sharing most of the metabolic and cellular pathways, and its simplicity in molecular and genetic manipulations make yeast particularly suited to produce human proteins for structure-function studies [35,36,37]. Indeed, yeast can perform many eukaryote-specific post-translational modifications such as proteolytic digestion, disulfide bridge formation, and some type of glycosylation [38]. However, differently from other eukaryotes, no trimming of mannose residues occurs in yeast [39]. Several yeast strains have been developed in which glycan remodeling has been induced, specifically deleting genes coding for glycosyltransferases and expressing the genes that carry out mammalian-specific glycosylation [40,41,42,43,44]. Indeed, DNA can be introduced or removed into/from the yeast chromosomes, enabling the creation of unique protein expression strains [45,46,47]. This “genetic” approach is largely used in yeast by exploiting homologous recombination: yeast transformation triggers the recombination between yeast sequences carried on the plasmid and homologous sequences in the yeast genome. This approach can serve different aims (Figure 1).

### 2.1. The Homologous Recombination

**Figure 2:**
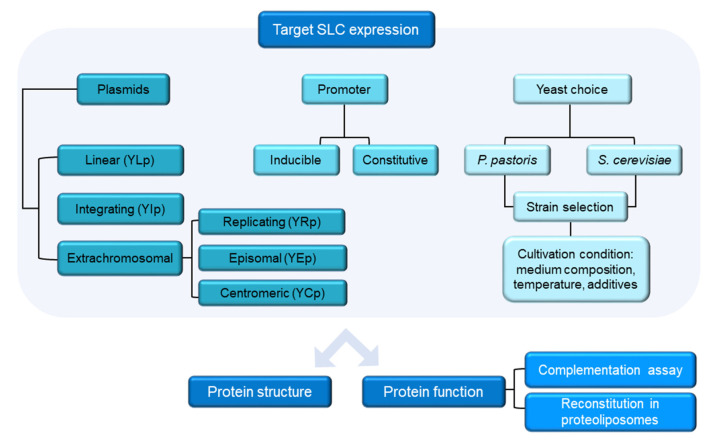
Workflow of a SLC production in yeast for structural and functional studies.

The observation that linear DNA fragments can efficiently promote recombination in *S. cerevisiae* led to the development of several methods for DNA manipulation in yeast [48]. Transformation has been used to clone genes by genetic complementation [49], to clone functional chromosomal components such as origins of replication [50], centromeres [51], and telomeres [52], and to clone functional suppressors acting as dominant negative mutants [53]. Recombination-based DNA manipulation methods also include integrative DNA transformation, which is used to induce gene deletion [54], and allele rescue which involves the transplacement of a mutation from the chromosome onto a plasmid-derived copy of that gene (Table 1) [55]. Indeed, homologous recombination in *Saccharomyces cerevisiae* can be considered as an easy and highly efficient cloning alternative. Yeast recombination cloning allows in a single step the assembly of multiple DNA fragments, deriving for example from many PCR reactions [56] (Figure 1). It is extremely efficient, requiring only 29 nucleotides of overlapping sequences that can be added to the synthesized oligonucleotides. Starting from these observations, by exploiting homologous recombination a yeast cloning cassette was used to point out a versatile method called any-gene-any-plasmid (AGAP) allowing the cloning of any gene (or combination of DNA fragments) into any vector in a single step without the need for ligase or a molecular cloning kit [57]. Towards the aim of obtaining the expression of a membrane transporter different aspects must be taken into account to transform yeast with the gene of interest (Figure 2).

### 2.2. Vector Choice

A yeast vector may exist as a self-replicating particle, independently of the yeast genome, or be stably integrated into the yeast genome. This last case should be considered to reduce expression fluctuations. Moreover, a chromosomally borne transgene confers the advantage of a uniform cell population containing the transgene. In contrast, plasmid numbers vary from cell-to-cell, with as many as 50% of cells in a culture under selection lacking the plasmid. Yeast vectors can be grouped into five general classes, based on their mode of replication in yeast: YIp, YRp, YCp, YEp, and YLp plasmids. Except for the YLp plasmids (yeast linear plasmids), all these plasmids can be maintained in *E. coli* as well as in *S. cerevisiae* and thus are referred to as shuttle vectors. Indeed, they contain two types of selectable genes: plasmid-encoded drug resistance genes for bacterial selection and a yeast gene which acts as dominant selectable marker only when the recipient yeast cell has a recessive mutation in the corresponding chromosomal copy of the cloned gene (See Section 2.3) [106]. Yeast integrating plasmids (YIp) contain selectable yeast genes but lack sequences that allow autonomous replication of the plasmid in yeast. The frequency of transformation of YIp plasmids is only 1 to 10 transformants/μg DNA, but transformation frequency can be increased 10- to 1000-fold by linearizing the plasmid within yeast sequences that are homologous to the intended site of integration on the yeast chromosome [106]. Besides integrating vectors, there are three classes of circular yeast plasmids characterized by extrachromosomal autonomous replication sequences (ARS) which confer the ability to replicate autonomously: YRp (yeast replicating plasmids), YCp (yeast centromeric plasmids), and YEp (yeast episomal plasmids). YRp plasmids have high frequencies of transformation (10^3^ to 10^4^ transformants/μg DNA), but transformants are very unstable both mitotically and meiotically. YRp plasmids can be present in high copy number (up to 100 copies per plasmid-bearing cell, although the average copy number per cell is 1 to 10). Introducing DNA portion from yeast centromeres into YRp-generated YCp plasmids allows an increase in plasmid stability during mitosis and meiosis. These plasmids (present in 1 to 2 copies per cell), have a loss rate of approximately 1% per generation. The increase in plasmid stability is fundamental for allowing protein production. The YEp vectors contain sequences from a naturally occurring yeast plasmid called the 2 μ circle and confer high transformation frequencies (∼10^4^ to 10^5^ transformants/μg DNA). Since they are propagated quite stably through mitosis and meiosis in high copy number, these plasmids are commonly used for high-level gene expression in yeast [106]. YLp (yeast linear plasmids) contain G-rich repeated sequences at their termini which act as telomeres and allow the plasmid to replicate as a linear molecule. They are highly unstable due to random segregation during mitosis. A crucial factor that drastically influences transcription and consequently translation rate is the promoter region. Commonly used promoters can be divided in two main classes: constitutive and inducible promoters (Table 2). Constitutive promoters lead to stable expression levels across varying culture conditions, while ‘dynamic’ or inducible promoters drive huge changes in expression level in response to specific stimuli [107] (Table 2).

Normally, constitutive strong promoters are used for biotechnological application to produce high levels of non-toxic target. Sometimes, the constitutive expression of certain proteins can be detrimental to cell growth due to product toxicity and the imposed metabolic burden [123]. To overcome this problem, several attempts at promoter engineering have been made aimed at tuning gene expression for biotechnological application [124].

### 2.3. Selection Strategies

The original and most commonly used strategy for the selection of transformed yeast cells exploits the auxotrophic markers: TRP1 [125], HIS3 [126], LEU2, URA3 [127], MET15, and ADE2 [128]. Expression plasmids are paired with available *S. cerevisiae* strains that are auxotrophic for tryptophan, histidine, leucine, uracil, methionine, and adenosine, respectively, by carrying full or functional knock-outs of these auxotrophic genes [129]. Besides auxotrophic markers, antibiotics can also be used as an alternative selection tool in a rich growth medium. However, since antibiotics affect ribosome function, expression studies should be performed in liquid media lacking antibiotics [129]. In the case of *P. pastoris,* the transformant selection is mostly conducted by using the antibiotic Zeocin, a member of the bleomycin/phleomycin family isolated from *Streptomyces*. Thus, clones deriving from multiple integration events can be selected increasing Zeocin concentration.

### 2.4. Yeast Transformation Methods

Transformation is the process by which exogenous DNA is introduced into a cell, resulting in an inheritable change or genetic modification [130]. Several methods have been described for transforming yeast cells using both biological and physical methods, and their efficiency has been improved over time [131,132,133,134,135,136,137,138]. The process depends on endocytotic membrane invagination and cell wall structure alterations [139]. Among the plethora of possibilities, the three most known methods are: electroporation [138], lithium acetate [135], and spheroplasts methods [133]. Despite the good transformation frequency, the spheroplast method is not employed, probably because of the tedious and complex procedure. The lithium method was published in 1983 [140] and is so far considered the method of choice for the transformation of *S. cerevisiae*. The reason for the effectiveness of this monovalent cation might be attributed to its mild chaotropic effect during the transformation. Lithium acetate (LiAc) was found to be 1.7-fold more effective than lithium chloride (LiCl). Importantly, lithium was not the sole contributor to the transformation of intact cells: incubation of intact cells with polyethylene glycol (PEG) and plasmid DNA is essential for transformation, a short heat shock at 42 °C of intact cells with PEG and plasmid DNA enhances the transformation efficiency, and transformation of the cells is most effective at the mid-log phase (OD610 = 1.6) [140]. The original protocol has been modified improving the efficiency to 5 × 10^6^–1 × 10^7^/μg of plasmid DNA from 10^8^ cells by immediately mixing washed intact cells with PEG, LiAc, plasmid DNA and single-stranded carrier DNA and incubating them at 42 °C for 40–60 min without pretreatment [135]. Electroporation was firstly used to transform intact *S. cerevisiae* cells by Hashimoto et al. [141]. Then, it started to be used as a general protocol for yeast transformation. The optimal conditions were voltage of 900 V, electroporation of early log-phase cells (OD600 = 0.3–1.0) in the presence of about 0.1 μg of plasmid DNA for high transformation efficiency. The transformation efficiency was increased by adding 1 M sorbitol up to 2–5 × 10^5^ transformants/μg of plasmid DNA [142]. Furthermore, it has been demonstrated that the pre-incubation of cells in the presence of both 100 mM LiAc and 10 mM dithiothreitol (DTT) improved the transformation efficiency by one to two orders of magnitude [137]. In the case of *P. pastoris* transformation, electroporation has become the election method.

### 2.5. Saccharomyces Cerevisiae

*S. cerevisiae*, also known as brewer’s or baker’s yeast, is a single-celled, budding yeast, approximately 5–10 μm in size. *S. cerevisiae*, unlike most other microorganisms, has both a stable haploid and a stable diploid state. Thus, recessive mutations are conveniently manifested in haploid strains, whereas complementation tests can be carried out by mating two haploids to get a diploid and checking its phenotype [143]. Being “generally recognized as safe” (GRAS), *S. cerevisiae* is a widely used model organism for diverse research, biotechnological and pharmacological applications, including the production of antibodies [144,145], G-protein-coupled receptors, transporters [146,147], and various therapeutic proteins approved by FDA [148]. *S. cerevisiae* can grow both aerobically and anaerobically on a variety of carbon sources and is able to use ammonia or urea as a nitrogen source. It also requires sulfur and phosphorus in its growth media [26], with a doubling time of about 1.5–2.5 h at 30 °C [149]. Metals such as calcium, iron, magnesium, and zinc enhance its growth [150]. In most eukaryotes, oxygen depletion triggers the switch from a respiratory to a fermentative metabolism, while in *S. cerevisiae* this switch can occur in response to a change in the external concentration of a fermentable carbon source such as glucose. The growth curve of yeast cultured in the aerobic batch is characterized by a biphasic pattern. In the first respiro-fermentative phase, most of the glucose is converted to ethanol, which is subsequently metabolized to produce CO_2_ and water in the second phase [26]. This ability to suppress the respiratory energy metabolism in the presence of oxygen in favor of aerobic alcoholic fermentation at high growth rates is known as the Crabtree effect [151]. While ethanol is toxic for most microorganisms, *S. cerevisiae* tolerates high concentrations (some strains up to 14%) [152]. Recombinant protein yields are usually highest before yeast cells reach the end of this respiro-fermentative phase, before the “diauxic shift” [26]. Consequently, *S. cerevisiae* cells are normally harvested just before this diauxic shift in a protein production experiment. In order to improve recombinant protein expression, a fully respiratory strain of *S. cerevisiae* has been created replacing the endogenous hexose transporters with a single chimeric transporter comprised of the first 6 transmembrane domains of the low-affinity Hxt1 transporter and the last 6 transmembrane domains of the high-affinity Hxt7 transporter [153]. The resultant TM6* strain was found to exhibit a respiratory behavior, most likely due to restricted glucose consumption and hence a reduced glycolytic rate [153]. Even the yield per cell of the expressed protein remained the same as wt yeast and the TM6* strain produced about 2–3 times more biomass, thus increasing the amount of the target protein [147]. One of the areas in which *S. cerevisiae* has proven to be an especially fruitful model is the area of mitochondrial research [154]. Indeed, the high similarity between yeast and human mitochondrial biogenesis and function renders *S. cerevisiae* an excellent model for studying human mitochondrial physiopathology [154,155,156,157]. Systematic approaches have been performed to optimize *S. cerevisiae* for recombinant membrane protein production, with the specific goal of understanding the molecular barriers to achieve high yields [158]. Using transcriptome arrays, the changes in mRNAs which occurred in different growth conditions (leading to low and high protein yields) were examined. Genes that were down-regulated under low-yield conditions were up-regulated under high-yield conditions and vice versa [159]. This approach led to the identification of genes that influence the yield per cell [160]: three are known components of the transcriptional SAGA (*GCN5* and *SPT3*) and mediator (*SRB5*) complexes, while a fourth, *BMS1*, is involved in ribosome biogenesis [159]. Thus, it was possible to maximize protein yield by increasing the *BMS1* transcript number [159].

### 2.6. Pichia Pastoris

The methylotrophic yeast *Pichia pastoris*, currently reclassified as *Komagataella pastoris*, can be considered a workhorse for biotechnological purposes, especially for heterologous protein production [161,162]. Recently, several *Pichia* strains have been engineered to generate more complex and more homogeneous N-glycosylation [163]. Moreover, the cholesterol-producing *P. pastoris* strain has been developed through four genetic modifications: *ERG5* and *ERG6* genes, involved in ergosterol biosynthesis, were replaced by homologous recombination with *DHCR7* and *DHCR24* genes allowing the synthesis of cholesterol [164]. One of the reasons for its high use in membrane protein production is that *P. pastoris* has a respiratory metabolism and can be cultured to exceptionally high cell densities (hundreds of grams per liter) on glycerol-containing media [26]. Thus, obtaining extremely high biomass obviates the need to achieve high over-expression levels that can trigger protein aggregation. *P. pastoris* assimilates methanol, due to the presence of two endogenous copies of the AOX gene coding for alcohol oxidase: AOX1 and AOX2 accounting for about 90% and 10% of the enzyme, respectively. Thus, by exploiting homologous recombination to replace with a gene of interest the AOX1 gene which is under the control of the AOX1 promoter, it is possible to induce/control protein expression. P_AOX_ is a promoter characterized by tight regulation and exceptional strength which is repressed in the presence of glucose or glycerol but strongly activated in the presence of methanol (Table 2) [27]. The *P. pastoris* vectors usually integrate into the host cell genome to produce a stably expressing clone. It is not possible to control the integrated number of copies that must be experimentally determined, either by real-time PCR [165] or through colony screening in the presence of increasing concentration of antibiotics [166,167]. It has been shown that strains with multiple copies of an expression vector from a single-transformed cell line can be selected by plating cells on agar media containing a higher concentration of the selection drug long after the original transformation [167]. These ‘jackpot’ clones with >10 copies of the expression vector had a proportional increase in recombinant protein [167]. In the case of cells transformed with just one or a few vector copies, a new selection can be made at high levels of drug long after the transformation thus obtaining the selection of clones characterized by increased copy numbers of the vector. This process, named post-transformational vector amplification (PTVA), resulted in cells containing multiple head-to-tail copies of the vector integrated at a single locus in the genome which would allow a proportional increase in recombinant protein [167]. However, there is not always a linear correlation between copy number and protein titer or quality [168]; thus the overall functional expression levels need to be experimentally assessed [27]. Expression screening allows identification of the best recombinant strain in terms of transporter expression. To this aim, the fusion to fluorescent reporters such as GFP can be employed [169]. Indeed, a fluorescent induction plate-screening assay would allow for the rapid detection of ”the best” clones [170].

#### Strategies for Proteins Production in *P. pastoris*

Protein production in *Pichia* cells is achieved in three standard stages (three-phase strategy) [171]. The first is a *batch phase* in which cells are grown in a basal medium containing glycerol as the sole carbon source. The excess glycerol represses AOX1 promoter and thus the expression of the gene under its control [172]. However, recent research has identified protein expression occurring during the pre-induction phase in cultures grown in bioreactors but not in shaker flasks indicating that the promoter is leaky under certain conditions [173]. During the second phase, the *derepression,* glycerol is fed in a limiting manner to increase cell density [174]. Finally, the expression of recombinant protein is induced by the growth-limiting addition of methanol. The duration of the *induction phase* may differ among membrane proteins, e.g., 24–96 h [164]. Besides the three-phase strategy, a biphasic production process has been proposed [175]: during the first step, *Pichia* cells grow in the promoter-repressing media containing glycerol or glucose; once high cell density is reached, this step is followed by media swapping for the subsequent and final methanol induction step. Removal of residual glycerol is mandatory to avoid inhibition of the AOX1 promoter (creating a derepressed condition) before adding methanol. An alternative high throughput method of inducing AOX1-based protein expression without replacing the entire medium has been described [176]. An auto-induction method has been developed, as well, using a buffered extra-YNB Glycerol Methanol (BYGM) auto-induction media to substitute the media-swapping method [28]. Expression levels of the three tested membrane proteins were comparable between BYGM (auto-induction method) and BMG/BMM (traditional/manual induction method). On the basis of the considerable safety risk associated to the toxic and flammable methanol and considering the important amount required, a method to make existing pAOX1-based production strains independent from methanol induction has been pointed out through the conversion cassette strategy [175]. Starting with the knowledge that maximum promoter activity is often governed by the levels of several TFs, this strategy is based on over-expression, under conditions where promoter activity is not repressed, of a transcriptional regulator able to upregulate pAOX1; this would allow modifying a strain in a glucose/glycerol regulated and methanol-independent system. Factor over-expression when the repressing carbon source is depleted (derepressed expression) would activate the *P. pastoris* AOX1 promoter mimicking methanol induction. Starting from a transformed *P. pastoris* strain, yeast cells are further transformed with another linearized plasmid containing a conversion cassette with the gene coding for the activator under the control of the promoter of the peroxisomal catalase 1 gene, pCAT1. This system would achieve a yield of between 7% and 44% of methanol-induced levels under small scale screening conditions without supplying additional carbon source during the derepression phase. Another critical parameter during *P. pastoris* cultivation on shaking flasks is appropriate oxygenation that is normally obtained by vigorous stirring, which triggers foam production. The addition of chemical antifoaming agents can prevent this phenomenon [177]. An alternative with respect to shaking flasks is represented by the use of a bioreactor which enables tight control of oxygenation, pH, and temperature, leading to an increase of the production of the target protein as in the case of SLC35A1 [158,178]. Numerous publications of guides and protocols highlight the importance assigned to *Pichia pastoris* in the production of membrane proteins among which are transporters [31,32,179], even though the choice of this yeast is linked to its ability to efficiently secrete correctly folded heterologous proteins which facilitate purification and downstream processing such as reconstitution studies in proteoliposomes [30,58,180,181].

## 3. Current Methodology for Purification and Functional Studies of SLCs

Protein expression represents the starting point for subsequent structure/function studies or high-throughput ligand screening. Interestingly, the deep knowledge of yeast metabolism and the availability of yeast strains deleted for specific endogenous transporters has made yeast a suitable system for studying transport in intact cells. On the other side, the expression of membrane proteins inserted in the yeast membranes allows the solubilizing of the proteins in a possible functionally active state and then purifying and performing functional studies in a reconstituted system. Biochemical and structural characterization of membrane proteins strictly depends on the ability to over-express and solubilize SLCs from natural lipid environments. SMALPs are a set of lipid particles (LPs) formed by the insertion of styrene maleic acid copolymers (SMAs) into a lipid bilayer. SMAs are amphipathic due to their mixture of hydrophobic styrene and charged, hydrophilic maleic acid moieties. Thus, they can be used for extracting proteins retaining the lipids present in the native membranes avoiding any exposure to a detergent that can have a negative impact on their function or structure determination. Moreover, preserving the native lipid composition can be crucial for a detailed understanding of the structure-function relationship [182]. Despite the advantages of this technique, there are only few examples of structural studies on membrane proteins using this approach, none of which ranges to the SLC superfamily [183,184,185]. Several examples of human SLC transporters expressed in prokaryotic expression hosts, purified in strong denaturing conditions, and on-column refolded are reported [23,186,187,188]. However, the isolation of a target protein from yeast membranes could be advantageous with respect to its recovery from inclusion bodies obtained following bacterial expression. Indeed, the use of mild detergents for the extraction of the membrane protein from the “native” lipidic environment, followed by on-column affinity chromatography, could facilitate biochemical and biophysical characterization.

### 3.1. Purification Strategies

To obtain purified proteins, the choice of the affinity tag added during the cloning procedure, according to the downstream applications, could be crucial. One of the most commonly used affinity tags used for the purification of human SLC transporters expressed in yeast is the polyhistidine tag (His-tag), consisting of 6His [30,58], 8His [103,180,189] or 10His residues [93,190]. During purification, there is the risk that the tag is inaccessible for binding to the affinity resin; thus, a linker between the tag and the target protein or a longer version of the His-tag may solve this problem. Furthermore, the tag position (N- or C-terminal) can be varied for better purification results. Immobilized metal ion affinity chromatography (IMAC) is commonly used in protocols exploiting the His tag. The advantage of using the poli-His tag consists in its small size, which could not hamper the subsequent functional and structural studies. Another tag commonly used for purification is the strep-II tag which consists of a Trp-Ser-His-Pro-Gln-Phe-Glu-Lys sequence [191]. This tag binds with high affinity to the streptactin resin, and its relatively small size may be beneficial in protein crystallization trials [192]. An alternative small tag is the HA tag which was useful for the production of several human SLC transporters in yeast [59,62,68,69,99,103,180,193]. Interestingly, in the case of the human CTR1 transporter, the metal-binding domain, naturally present in the protein, was exploited for IMAC purification [194]. In case the tag hampers structural studies, it is possible to remove it by introducing a protease cleavage site between the tag and the target protein during cloning. A further strategy for improving crystallization trials is the mutation of residues involved in N-glycosylation starting from the assumption that glycosylation may hamper crystal formation [19,194].

### 3.2. From Complementation Assays to Biochemical Characterization

The yeast *Saccharomyces cerevisiae* provides a powerful tool for the functional analysis of human genes. By using yeast strains knock-out for a specific gene or group of genes involved in the transport or metabolism of sugars, amino acids, nucleotides, and other endogenous compounds, it is possible to monitor the restoration of transport activity following yeast transformation and human ortholog expression. According to this idea, a pioneering experiment was conducted to create a strain in which all genes encoding for transporters with hexose uptake activity, such as *HXT1-17*, *AGT1*, *MPH2*, *MPH3*, were deleted [65]. The strain, designated as hexose transporter-deficient (*Hxt^0^*, EBY.VW4000), is not able to grow on media with glucose, fructose or mannose as the sole carbon source and grows only very slowly on galactose (Table 1). For maintenance, the strain is routinely cultivated on maltose, a disaccharide that is taken up through the specialized maltose symporters encoded by the MALx1 locus. Thus, the hxt^0^ strain offers an excellent opportunity to clone and characterize heterologous hexose transporters by replacing the function of endogenous transporters [65]. A focus on this strategy for the study of the GLUT transporter family was done in Section 4.2. By exploiting the same approach, a huge number of membrane transporters have been investigated, obtaining information about function and kinetics (specificity, mechanism of transport, etc.), the role of specific residues revealed to be critical for mechanism or specificity, and pathological variants. Moreover, this strategy allowed inhibition studies and identification of human and yeast transporters. A summary of the procedures pointed out for purification and transport studies of SLC human transporters expressed in yeast has been reported in Table 3.

#### 3.2.1. Sophisticated Complementation Assays

More complex assays have been pointed out for the study of some members of the SLC25 family, the mitochondrial carriers, among which are the phosphate–carrier PiC (SLC25A3) or the ADP/ATP carriers, *ANT*s (SLC25A4, 5, 6, and 31). Their expression has been confirmed, taking advantage of the fact that their activity is not essential for cell growth under fermentation culture conditions but becomes essential under non-fermentation conditions such as growth on a glycerol medium that requires a functional mitochondrial respiratory system. Indeed, in a non-fermentable medium, net ATP can only be generated in mitochondria through OXPHOS [72,73,74,75,213,217].

Another more sophisticated complementation assay has been pointed out for members of the nucleoside transporters families, SLC28 and 29. The CNTs catalyze Na^+^-dependent symport of nucleosides against their concentration gradients, whereas the ENTs facilitate bidirectional transport of nucleosides down their concentration gradients. Yeast lacks the mechanisms for thymidine transport and phosphorylation to dTMP. Thus, dTMP production from dUMP is the only way for growth to take place. Thus, inhibition of this process, achieved using methotrexate (MTX) and sulfanilamide (SAA), would block cell growth. On the contrary, in yeast made competent for thymidine phosphorylation, in the presence of MTX and SAA, the expression of a heterologous thymidine transporter would restore growth. Cells were grown under dTMP starvation but in the presence of thymidine showing that the expressed hSLC complemented the imposed thymidylate depletion in *S. cerevisiae* by transporting thymidine [89]. This confirmed the production of the nucleoside transporters and their correct targeting to the yeast cell surface. A double-permease knock-out strain lacking the endogenous uracil permease (*FUR4*) and uridine permease (*FUI1*), was employed to express, for example, hCNT2 [90]. Survival in 5-fluorouridine (FUrd) indicated the successful integration of the target gene into the yeast genome, with disruption of the *fui1* gene, considering that FUI1 mediated transport of FUrd led to yeast death.

#### 3.2.2. Protein Targeting and Investigation on Specific Organelle Functions/Processes

The targeting of proteins in specific membranes, like mitochondrial membranes, has allowed specific organelle functions/processes studies in the isolated organelles or vesicles. This is the case of mitochondrial transporters like the UCPs, PNC, or MCART1. The hUCP1 is known to dissipate metabolic energy as heat by a controlled uncoupling of the oxidative phosphorylation process: coupling between respiration and oxidative phosphorylation is dependent upon a proton gradient across the mitochondrial membrane which can be impaired by UCPs. Mitochondria, in which protein targeting was confirmed, were isolated from the yeast, and bioenergetics were evaluated in terms of oxidative phosphorylation, mitochondrial membrane potential, respiratory chain activity (see Table 3). Concerning studies in sub-cellular components of the yeast cell, some examples about yeast vesicles are reported in Table 3.

In some cases, the expressed transporter does not reach the correct destination completely or at all. An example is the SLC4A1 transporter for which two different isoforms are described: the full-length erythrocyte variant (eAE1) and the kidney variant (kAE1) lacking the first 65 N-terminal amino acids present in the erythrocyte isoform [67]. Concerning the kidney variant kAE1, trafficking studies revealed that only a part of the transporter molecules efficiently enters the secretory pathway reaching the plasma membrane, the rest being accumulated in other membranes. The correctly targeted protein was revealed to be functional in the *S. cerevisiae* strain BY4742 cells [59]. The trafficking was also studied employing two different yeast strains, defective either in endocytosis (*Δend3* mutant) or in vacuolar degradation (*Δpep4* mutant). Full-length variants and HA-tagged kAE1 reached the yeast plasma membrane even though at a minor extent; indeed, most of the transporter molecules accumulated in intracellular membranes. The eGFP-tagged kAE1 (GFP-KAE1) was produced to deeply investigate if the accumulation of kAE1 in membranes different from plasma membranes only occurred in the ER or also in Golgi or endosomal structures [69]. Co-localization studies with different organelle markers were performed using the wild-type BY4742 cells expressing the wild-type eGFP-tagged kAE1 (GFP-KAE1). The N-terminal addition of eGFP has been demonstrated to not affect kAE1 targeting to the yeast cell surface. Using the truncated kAE1 variants, the C-terminal region of the transporter has been identified as crucial for the undesired accumulation and activation of the cellular response to unfolded proteins (UPR). By testing the additional overexpression of different ER proteins involved in protein folding, it has been demonstrated that the increase in ER folding capacity would prevent the unfolded protein response (UPR) allowing the protein to reach the plasma membrane. The BY4742 GEV cells under the control of a β-estradiol-inducible GEV promoter system, which allowed a dose-dependent regulation of kAE1 expression, were employed. The use of strong promoters and multicopy vectors that exceed the folding capacity of yeast was revealed to be the cause of aggregation and accumulation. Another example is given by SLC9, hNHE1 which accumulated in the plasma membrane three times higher at 15 °C compared to 30 °C, reaching a density of 0.7% (*w*/*w*) in the crude membranes 72 h after induction. Cell culture was precooled before 20% (*w*/*v*) galactose induction in the absence of glucose. The produced protein, accumulated with the expected molecular weight, maintained a folded structure when purified and degradation was reduced [62].

#### 3.2.3. Study of Pathological SLC Variants

The yeast system allows to assay variant functionality as well. To this aim, mutations associated with adPEO, a mitochondrial disorder linked to specific mutations (A114P and V289M), have been introduced into *hANC1.* The expressed mutants were not able to restore yeast growth on the non-fermentable carbon source [75]. Another example is represented by mutations in the *AGC2* gene which result in two age-dependent disorders, the neonatal intrahepatic cholestasis which is caused by citrin deficiency (NICCD), and the adult-onset type II citrullinemia (CTLN2) [80]. Variant expressions reduced cell growth, confirming that mutations impair AGC2 function. Moreover, *S. cerevisiae* has been employed to characterize the function of the ornithine transporter ORNT2 (SLC25A16) and to test the pathogenicity of the *ORNT1* (*SLC25A15*) mutations found in hyperornithinemia–hyperammonemia–homocitrullinuria (HHH) patients. To this aim, expression of these genes was achieved in the *ΔArg11* Y02386 strain. Deletion of *Arg11*, corresponding to the *S. cerevisiae ORNT1* orthologous, rendered these cells auxotrophic for arginine. Differently to *ORNT1*, which could complement the deletion of *Arg11*, *ORNT2* did not restore the ability to grow in a selective medium lacking arginine. Three of the residues that diverged in ORNT2 with respect to ORNT1 were replaced with the corresponding amino acids present in ORNT1. The replacement allowed us to correct the *ΔArg11* phenotype. The crucial role of arginine 179 was elucidated which likely affects the substrate-binding pocket altering the affinity for ornithine. All the disease-causing alleles had a detrimental effect on the ability of the hORNT1 to complement the deletion of *Arg11* [81]. Then, mutated TMEM165 (SLC64A1) has been expressed in *S. cerevisiae* strain deleted of the yeast Golgi Ca^2+^ and Mn^2+^ transporters, *Gdt1p* and *Pmr1* [105]. Direct evidence of calcium and manganese transport by TMEM165 has been provided. Yeast cells were used for calcium and manganese sensitivity assays (drop tests), to explore the impact of disease-causing mutations on transport activity.

#### 3.2.4. Transporter/Drug Interactions

Some examples are reported concerning SLC/drug interaction studies in yeast. The wild type hENT1 and the N glycosylation-defective mutant (hENT1/N48Q) have been expressed in *S. cerevisiae*. Replacement of an Asn48 with Gln increased the apparent affinity of the transporter for dilazep and dipyridamole [223]. GFP-tagged wild-type hENT1 and hENT1 mutants were tested for their sensitivity to the 5-fluorouridine and for tubercidin toxicity which was reversed by NBMPR. Substitution of glycine 154 in hENT1 with the corresponding serine of hENT2, converted hENT1 to a transporter that exhibited partial characteristics of hENT2 in terms of sensitivity towards the inhibitors NBMPR, dilazep [221]. Radiolabeled nucleosides such as [^3^H]adenosine were used in transport assays in the absence or presence of the potential substrate/inhibitors. A strain carrying a mutation in the *ADE2* gene which does not allow survival in the absence of adenine but only if external adenosine is transported into the cell by a functional nucleoside transporter, such as hENT1 (adenosine rescue), was employed. Following adenosine transport, the mutation in L92 was found to alter guanosine and the sensitivity to NBMPR and dilazep [94]. A further mutation in hENT1 (hENT1-M33I) reduced sensitivity to dilazep and dipyridamole with respect to the wild type hENT1y [224]. hENT2-I33M was created which showed higher dilazep and dipyridamole sensitivity and higher uridine affinity compared with wild type hENT2. These findings allowed the identification of a common region of inhibitor interaction [222].

Another example is given by the thiamine transporter SLC19A3 expressed in a strain in which thiamine transporters genes THI7 and THI3 were deleted [71]. The antimalarial chloroquine has been found to inhibit it. Another example is the hSV2A (SLC22B1) expressed in the EBY.VW4000 strain. In this system, it was revealed to be a galactose transporter [212]. Galactose transport was inhibited by the antiepileptic drug levetiracetam which specifically binds to SV2A. Yeast-based HTS strategies have been pointed out to identify compounds that inhibit GLUTs (see Section 4.2 for details), ANTs, nucleoside transporters. Closantel and CD437 were identified as broad-spectrum ANT inhibitors, whereas leelamine was found to be a modulator of ANT function [213]. The inhibitory effect of different drugs was investigated on both the concentrative and equilibrative transporters. The ability of azacitidine, decitabine, and gemcitabine to inhibit the uptake of [^3^H]uridine was investigated in *S. cerevisiae* cells transformed with pYPhENT1, pYPhENT2, pYPhCNT1, pYPhCNT2, or pYPhCNT3 [218]. hCNT1 and hCNT3 showed high apparent affinities for these inhibitors, differently from other hNTs. Interaction of the BCR-ABL tyrosine kinase inhibitors bosutinib, dasatinib, imatinib, nilotinib, and ponatinib was assessed, as well, investigating their effect on [^3^H]uridine transport [219]. TKIs inhibited transport to different extents. Moreover, TKIs inhibited uridine uptake competitively in hENT1-producing yeast cells. The effect of the purine nucleoside analogs, clofarabine, cladribine, fludarabine on hENT1, hENT2, and hCNT3 was investigated [233]. Inhibition of [^3^H]adenosine uptake by Cl-F-ara-A, Cl-dAdo, and F-ara-A was measured and the relative binding affinities were calculated. The transporters all exhibited higher affinities for clofarabine than for either cladribine or fludarabine. hCNT3 was tested for transport of the purine nucleoside analogs, clofarabine, cladribine, fludarabine [233]. Higher affinity for clofarabine was found. Uptake of [^3^H]clofarabine and [^3^H]adenosine into yeast was measured and the relative binding affinities were calculated measuring the drug’s ability to inhibit [^3^H]adenosine transport.

The activity of PEPT1 (SLC15A1) was investigated in peptide uptake assays performed exploiting ^3^H-D-Phe-Ala by the rapid filtration technique [63,209]. Inhibition of D-Phe-Ala uptake by different compounds was performed including β-lactam antibiotics. Moreover, ligands useful for photodynamic therapy were tested [234]. Uptake of the radiolabeled model dipeptide glycylsarcosine (GlySar) was measured in transport assays performed in yeast cells and hPEPT1 inhibition by the antiviral prodrug, oseltamivir was observed [210]. [^14^C]Oseltamivir was employed as well revealing it is not a substrate for PEPT1. [^3^H]GlySar uptake and [^3^H]Cefadroxil uptake were investigated for hPEPT2 [211].

#### 3.2.5. Biotechnological Implementation

A transporter engineering strategy has allowed us to solve biofuel toxicity due to fatty alcohol storage inside the cells after production. To increase the production of fatty alcohol to exploit as biofuel, the human transporter FATP1 (SLC27A1) able to perform fatty alcohol efflux, has been expressed in the high fatty alcohol production yeast strain, FOH33 [88]. FATP1 expression enabled yeast cells to achieve a faster growth rate and a higher final cell mass with respect to the control strain allowing to relieve the toxicity associated with the intracellular fatty alcohols.

## 4. Selected Case Studies

Employment of yeast for expression of a high amount of human transporters has allowed deep investigation of functional and/or structural features of the proteins. Here are reported case studies for which, starting from a detailed description of the expression conditions and the adopted purification techniques, an in-depth biochemical characterization has been described.

### 4.1. SLC1A5

ASCT2 (SLC1A5) has been over-expressed in *P. pastoris*. Two different recombinant constructs were used to transform the *Pichia* X33 strain, the wild type and the optimized one, defined as pPICZB-(wt)hASCT2-6His and pPICZB-(Opt)hASCT2-6His. Zeocin-based selection of putative multi-copy recombinants has been performed [30]. For large scale protein production, transformants were grown in a 3 L fermentor. *P. pastoris* has been employed for the over-expression of the hASCT2 Cys-mutants as well [196]. Each of the eight Cys residues was substituted with Ala, by site-directed mutagenesis and the mutant cDNAs were cloned in the pPICZB vector obtaining the pPICZB-ASCT2-6His mutant constructs that were linearized with *Pme*I and used to transform *P. pastoris*. WT and mutant protein expression were induced by 0.5% methanol. The growth in methanol was performed at 30 °C for three days adding fresh methanol every 24 h. At the end of the induction phase, the cultures were centrifuged, subject to lysis by high pressure and ultracentrifuged for obtaining the membrane fraction. 1.5 g of washed membranes (300 mg/mL) were solubilized for performing IMAC purification with a final yield of 10 mg of purified protein per Liter of cell culture [30]. In the case of the C363A mutant, the expression was obtained growing cells in BMMY medium at 20 °C for only one day.

Since its over-expression in *P. pastoris*, functional and structural knowledge on the ASCT2 transporter has increased considerably, clarifying the mechanism of transport [20,33,195,196,197,198,199,235]. Protein purification was performed on a Ni-NTA column by exploiting the 6His-tag. The preferred detergent for solubilization, C_12_E_8_, was proven by the functional characterization. Gel filtration chromatography by Superdex 200 showed a 150 kDa oligomeric form, suggesting that the protein tends to form trimers. [^3^H]-glutamine transport was followed in proteoliposomes reconstituted with the recombinant protein. Thus, the reconstitution procedure was optimized and a sodium dependent [^3^H]-glutamine/glutamine antiport was measured. The ASCT2 transporter showed asymmetric specificity for amino acids: Ala, Cys, Val, Met were only inwardly transported, while Gln, Ser, Asn, and Thr were transported bi-directionally. This finding suggests that hASCT2 provides cells with mostly neutral amino acids on the basis of metabolic needs of cells or the amino acid concentrations (balance of the intracellular amino acid pool). pH dependence was investigated, and an optimum at pH 7 was found. The effect of specific reagents on hASCT2 was investigated as well and glutamine transport inhibition was found in the presence of hydrophilic thiol specific reagents indicating the involvement of one or more cysteine residues in the mechanism of transport. In particular, Cys 395 was suggested to be involved considering that it is the only cysteine residue exposed towards the extracellular side of the protein and thus accessible to the hydrophilic reagents [30]. Protein orientation in liposomal membrane (sidedness) was investigated by reshuffling (freeze and thaw followed by pulse sonication): all proteoliposomes were sided similarly to the cell membrane. In the proteoliposome system a kinetic analysis was performed as well: internal and external Km values of hASCT2 for neutral amino acids were calculated. The difference between the external and internal Km values is in favor of the functional asymmetry of the transporter and in agreement with the structural asymmetry of the hASCT2. In addition, the kinetic mechanism of the transport was evaluated by a pseudo-bi-substrate kinetic analysis of the Na^+^-glutamine_ex_/glutamine_in_ transport reaction. A random simultaneous mechanism was found that correlated well with an oligomeric structure of the transporter; thus, cross-linking experiments confirmed the formation of a stable oligomeric functional state. By imposing a liposomal membrane potential, electrogenicity was investigated, showing a dependence on external Na^+^ [198]. Interestingly, further studies measuring transport of [^14^C]-Cysteine in proteoliposomes clarified its role. The proteoliposome approach allowed us to follow both uptake or efflux of Cys. On one hand, Cys acted as a strong competitive inhibitor by high-affinity binding; on the other hand, the same amino acid triggered, at slightly higher concentrations, substrate efflux by a uniport mode. Very probably, this action relies on binding of Cys to an additional site of the protein [197]. The first insights concerning the structure/function relationships of the human ASCT2 were gained in 2018 [196]: the reducing agent DTE was used to show that SH/S-S formation would regulate transporter activity, turning it on or off. This probably involves a different mobility for the elevator portion of the protein across the plasma membrane. To this aim, Cys mutants were produced (see above) and transport activity was measured. All mutants were functional, and the effect of DTE and Methyl-Hg was tested on each of them. Among all mutants, C467A was insensitive to reducing agents and to Methyl-Hg: the C467 residue, for which a much higher Km for glutamine but not for sodium was measured, was identified as part of the substrate binding site of hASCT2. Later the involvement of this Cys residue as a molecular determinant of the antiport mechanism has been revealed [33]. The C467A mutant functionally reconstituted in liposome showed, differently by the wild type protein, the unique ability to mediate a low measurable unidirectional glutamine transport which then can be considered as an unspecific phenomenon probably associated with a difference in conformational change among the two types of proteins. The role of C467 in the internal side of the substrate binding would be to facilitate the release of an H-bond. This bond would probably slow down the reorientation of the substrate-free transporter towards the outward conformation, according to the antiport mechanism. In the case of the mutant C467A, the lack of SH group generates a protein able to mediate a measurable unidirectional transport reaction.

Cholesterol interaction with protein has also been investigated. Cholesteryl hemisuccinate strongly activated the Na^+^_ex_-[^3^H]glutamine_ex_/glutamine_in_ antiport in proteoliposomes without affecting the Km for glutamine. A strategy based on the specific targeting of tryptophan and cysteine located in the neighborhood of cholesterol poses, which were revealed by computational approach, has been employed to reveal the direct binding [200].

Interestingly, the overexpressed protein reconstituted in the proteoliposome system allowed us to reveal not just neutral amino acid transport, but also the sodium dependent antiport glutamate/glutamine stimulated by a proton gradient. A proton flux directed towards the intraliposomal/cellular compartment coupled to glutamate has been shown [195]. Moreover, the role of sodium has been investigated. A role in stability and folding has been found and by setting up a fluorometric assay, an inwardly directed flux of sodium has been demonstrated with a stoichiometry of 2 Na^+^:1 Gln [199].

The protein obtained according to the procedure pointed out by Pingitore et al. was used for structure studies, as well. The resolution of the ASCT2 3D structures highlighted that the transport occurs with a one-gate elevator mechanism in which the transport domain slides over the scaffold domain with the consequent translocation of amino acids [20].

Considering ASCT2 over-expression in cancer cells, it has been proposed as a potential target for antitumor drugs, showing the requirement of a system/tool like yeast cells or liposomes to perform high-throughput screening.

### 4.2. SLC2A1, SLC2A2, SLC2A3, SLC2A4, SLC2A5

The SLC2 family includes fourteen hGLUT transporters, categorized into three clusters based on sequence similarity. SLC2A1, SLC2A2, SLC2A3, SLC2A4, and SLC2A5, which are representative members of the three clusters, have been expressed in yeast. The hGLUT1 (SLC2A1) wild-type and the His-tagged constructs were used to transform different *S. cerevisiae* strains: the proteinase deficient strains (BJ2168 and MC2) and the strain which can tolerate copper induction (DY150). Different vectors were tested to achieve the best protein expression level: pYPMA, pYES2, and pYEX-BX (see Table 2) [201]. Later, engineered *S. cerevisiae* strains, incapable of growing on glucose or related monosaccharides, were created generating useful assay systems for ligand screening of the class I GLUTs and of the class II member, GLUT5 [202,204,236]. The first generated strain was the EBY.18ga (*Δhxt*) which was deleted for all the endogenous hexose transporters HXTs, the homologous of the GLUTs; the EBY.VW4000 strain (*Δhxt Δmph*) was then obtained by further deletion of genes coding maltose transporters (*MPH2*, *MPH3*) through the loxP-Cre recombinase system in a CEN.PK2-1C strain background [64,65]. Detailed protocols are available for handling this strain and characterizing expressed sugar transporters [237]. These strains have been employed for the first time to achieve hGLUT1 and hGLUT4 expression. To obtain the insertion of the glucose transporters into the membrane in an active form, additional mutations had to be added to both the yeast genome and the GLUT sequence [64,236]. Thus, further strains were constructed: the EBY.S7 (*Δhxt fgy1-1*) and the EBY.F4-1 mutant strain (*Δhxt fgy1-1 fgy4X*) (see Table 1). Homologous recombination allowed *hGLUT1* and *hGLUT4* insertion in all these four yeast strains using the linearized YEp4H7 plasmid. Protein production was under the transcriptional control of a strong and constitutive HXT7 expression cassette. Only the successful expression of a functional protein would allow the cells to grow. This was achieved only in strains EBY.S7 and EBY.F4-1. The HXT2 promoter was tested in place of the strong HXT7 promoter in controlling the expression of GLUT4 in the strains EBY.S7 and EBY.F4-1. The slow growth of the EBY.S7 cells and much faster growth of the EBY.F4-cells were observed. Subcellular localization analysis by density gradient centrifugation revealed that GLUT1 localized at the plasma membrane of EBY.18ga and EBY S7 strains, while GLUT4 was in intracellular structures of EBY.18ga strain. Concerning GLUT4, maximal localization at the plasma membrane was found in EBY.F4-1 cells. Thus, kinetics was investigated in intact yeast cells using D-[U-^14^C]-glucose employing the EBY.F4-1 mutant strain for GLUT4 and EBY.S7 for GLUT1. Inhibition by cytochalasin B and substrate specificities were measured. Some years later, a further attempt to express hGLUT1, was performed employing the *S. cerevisiae* glucose transport-null strain, RE700A, in which just HXT 1–7 of the 20 hexose transporters were deleted and which was unable to grow on glucose media but capable to grow on maltose [68]. The simplest strain was transfected with the p426GPD yeast expression vector containing DNA encoding for the GLUT1 wild-type, the mutant GLUT1-338-A3, or the C-terminal hemagglutinin 6His-tagged GLUT1 (GLUT1-HA-H6). hGLUT1 localized to the plasma membrane. Indeed, this strain was able to grow on glucose. Transport assays were performed in cells following [^3^H]-D-glucose or [^3^H]-2-deoxy-D-glucose uptake. RE700A-GLUT1-338-3A grew on maltose but could not survive on glucose. Moreover, the 5′UTR-GLUT1-HA-H6 construct was obtained improving GLUT1 expression in RE700A. The choice of the RE700A strain was based on the consideration that repression by glucose is exerted on many of the hexose transporters. An upregulation under selective pressure has been emphasized [237], which could likely lead to a high frequency of suppressor mutations. These considerations make *hxt0* the election strain for GLUTs study. The *S. cerevisiae* EBYS7 strain was employed to investigate the role of Ile287 in human GLUT1. This residue corresponds to Asn331 of the yeast Hxt2 which has been shown responsible for the high-affinity glucose transport activity of this transporter [203]. To this aim the GLUT1-pVT construct has been prepared: human *GLUT1* gene has been introduced in the multicopy vector pVT102-U (YEp URA3 bla) under the control of the ADH1 promoter. 287X series of GLUT1 mutants were substituted for the corresponding region of GLUT1 in GLUT1-pVT. Glucose transport in cells has been measured following D-[^14^C]glucose uptake. “Medium-size” residues at the 287 position were determinants of high glucose affinity. Effects of phloretin and cytochalasin B were evaluated supporting the Ile287 location at or close to the presumed exofacial binding site for glucose. Human GLUT1 was expressed in *P. pastoris*, as well. To this aim, it was cloned in a pPICZ B vector and the X33 strain was employed [180]. An 8His tag and a Factor Xa cleavage site were inserted. The N-linked glycosylation site was removed to avoid impairments in protein crystallization. The following constructs were obtained: aglyco-GLUT1–FXa-polyHis and aglyco-GLUT1 D37–FXa-polyHis. The last was obtained by removing 37 AA at the C terminus to obtain a less flexible protein suitable for crystallization trials. Multiple copies of the expression cassette (pAOX1 promoter–gene–Zeocin) were integrated into the yeast genome by homologous recombination. GLUT1 was properly targeted to the plasma membrane. After purification, GLUT1 was functionally reconstituted into proteoliposomes.

Concerning the hGLUT2 expression, the C-terminally-fused to a Green Fluorescent Protein (GFP)-8His tag was expressed under the strong galactose inducible CYC-GAL promoter which was enhanced by over-expressing the Gal4 transcriptional activator in the PAP1500 strain [60]. The construct was generated by in vivo homologous recombination transforming PAP1500 cells with the hGLUT2 PCR fragment, a GFP PCR fragment, and the pEMBLyex4 vector (PAP7913). Growth at 15 °C resulted, after galactose induction, in a stable accumulation of the protein at the plasma membrane and intracellular membranes. Expression of functional hGLUT2 and hGLUT3 was achieved in the hxt0 yeast system [66]. The linearized p426H7 or pRS62K vector was assembled via homologous recombination into EBY.VW4000, EBY.S7 or SDY.022 with the GLUT3 which was amplified with oligonucleotides having 30–40 base pair overhangs to the applied promoter (HXT7-1–329) or terminator (CYC1) region. Growth was observed just in the EBY.S7 and SDY.022 cells after five days of incubation on glucose indicating that no further mutations, except for the *fgy1* mutation, in the yeast strain were essential for restoring growth on glucose. Notwithstanding, in the bigger colonies the S66Y mutation was found beneficial for functionality but not essential for growth differently by the *fgy1* mutation typical of the EBY-S7 strain. These findings were confirmed by employing the GLUT3-S66Y mutant. Concerning hGLUT2, no growth was observed. Comparison among GLUTs primary sequences drove mutagenesis of the amino acids 101–103. Moreover, N-glycosylation site at position 62 was mutated. None of these modifications resulted in growth on glucose containing medium. An important difference among GLUT2 and the other GLUTs is the presence of the largest extracellular loop between TMD1 and TMD2. Hence, a GLUT3-like loop was introduced obtaining the GLUT2Δloop. Moreover, other mutants were obtained in which a serine substituted the loop (GLUT2ΔloopS) to be comparable to GLUT3 and the lysine preceding the deleted region was mutated to serine (GLUT2K54S_Δloop), corresponding to S55 of GLUT3, but inserted at the beginning instead of the end of the deleted loop. Each of the loop modifications allowed the growth on glucose. A crucial effect on GLUT2 activity was played by the additional point mutation Q455R combined with the ΔloopS modification. The best activities of GLUT2 and GLUT3 constructs were observed in the EBY.S7 cells. The additional mutation in GLUT2ΔloopS (Q455R) and GLUT3 (S66Y) improved the transport activities. Inhibition of GLUT2 and GLUT3 by phloretin was found confirming this system’s applicability to screen GLUT2 inhibitors.

Functional expression of the human GLUT5 in the *hxt0* strain was achieved by the introduction of mutations [205]. The p426MET25 vector was used to transform EBY.VW4000 cells with the WT or the truncated form of the hGLUT5 (GLU5tr) in which the first seven amino acids at the N-terminus had been removed. Expression of GLUT5 should confer growth of yeast cells on fructose but a very low percentage of the clones could grow. In isolated plasmids, the following mutations located in the second transmembrane helix of GLUT5 (as occurred for GLUT1) were found: S72Y and S76I (in both GLUT5 and GLU5tr); S76N (in GLUT5). These mutant constructs were amplified and inserted in the pRS72K vector between the truncated HXT7 promoter and cyc terminator by a gap-repair procedure and transformed into EBY.VW4000 strain. Growth was confirmed on fructose-containing media. GFP constructs containing mutations were prepared: sGFP was inserted in the p426MET25_GLUT5 construct. Localization at the plasma membrane and endomembrane system was observed, indicating that mutations do not directly affect the trafficking of the protein. This system was employed to evaluate inhibitors or activators through the ^14^C-radiolabeled fructose uptake assay or by cell growth assays in fructose-based media. Mutations do not have a major influence on transport kinetics and inhibition.

Inhibitor discovery was undertaken through in silico ligand screening and in vivo validation in the *hxt0* yeast system [202,204]. 163 small compounds, selected by in silico ligand screening using a GLUT2 inward-facing conformation model, were tested in the EBY.S7 strain expressing the human GLUT2ΔloopS_Q455R which shares the GLUT2 functional properties [202]. ^14^C-hexoses (glucose or fructose) have been employed in transport assays. The selectivity of the identified GLUT2 inhibitors was determined, testing their effect on the other Class I GLUTs (GLUT1, 3, 4) and on the class II GLUT5. GLUT1, GLUT3-S66Y, GLUT4, and GLUT5-S72Y were inserted in specific hxt0 strains (EBY.VW4000 for GLUT5, EBY.S7 for GLUT1-3, and SDY.022 for GLUT4). Very potent and specific GLUT2 inhibitors were identified. 200 ligand candidates selected through in silico screening of an 8 million compounds library against the inward- and outward-facing models of GLUT3, were tested for in vivo inhibition of GLUT3 expressed in the *hxt0* yeast system [204]. The effect was evaluated by measuring the accumulation of radioactive glucose in *hxt0* yeast cells in the absence or presence of the compounds. IC50 for transport inhibition was calculated in the wild-type and S66Y GLUT3 (GLUT3-S66Y) *hxt0* yeast system. Specificity was assessed by testing the resulting six new GLUT3 inhibitors on the other GLUTs, employing strains and constructs previously described.

## 5. Discussion

The international scientific community strongly recommends improving the study and the de-orphanization process of the SLCs, most of which are known to be implicated in the development and progression of many diseases and the vast majority of which have not yet been considered in therapy [7,13]. Although the employment of human cells is gradually becoming established in human transporter production aimed at structural studies, by far the cheapest yeast remains highly topical as a transporter expression system. This is due to the various advantages associated with this system (for which the reader is referred to Section 1) and to the various opportunities which yeast offers. Many examples of transporters expressed in yeast, functionally and kinetically characterized by in vivo or in proteoliposomes transport assays, are reported in this review. Other cases of transporter expression have allowed SLC identification which has been made possible by the employment of engineered deleted strains: this is the case of the hCTR1 (see Section 3.1 for details), responsible for the high-affinity uptake of copper in humans, identified by complementation assay in yeast; or of the newly identified Golgi nucleotide sugar transporter SLC35B4 or of SLC25A51 (MCART1) (see Table 3 for details). Engineered deleted strains allowed yeast transporters identification as well, as occurred in the case of the yeast aspartate-glutamate carrier Agc1, identified as the orthologue of the human AGC isoforms, Aralar1 (SLC25A12/AGC1) and citrin (SLC25A13/AGC2) (see Table 3 for details). The yeast system has allowed the unraveling of the role played by residues conserved among transporters and the evaluation of the effect of disease-associated mutations on protein function (see as example Section 3.2.3 for details). Then, specific transporter inhibitors have been identified by the in silico ligand screening/yeast-based HTS strategy (see Section 3.2.4 and Section 4.2 for details). Yeast has been employed for the cysteine-accessibility and permeant protection assays, as well, which made it possible to postulate the translocation pathway (see Section 4.1 for details). In addition, yeast was revealed to be precious for industrial purposes: transporter engineering strategy in yeast had important effects on industrial production (see Section 3.2.5 for details). On the other side, problems that may be encountered using yeast are in some cases apparent, e.g., the lack of the correct localization of a transporter in the yeast plasma membrane which could be overcome by purifying and reconstituting the protein in liposomes for functional studies; moreover, strategies have been developed to increase ER folding capacity and thus to prevent the unfolded protein response (UPR), allowing the protein to reach plasma membrane (see Section 3.2.2 for details). Ultimately, the easy genetic manipulation and relatively low production costs make yeast a tool of excellence for protein production in general, and thanks to the ability of yeast to be inserted into membrane correctly folded membrane proteins, it continues to be a tool of choice for the production of human SLCs for subsequent studies of structure and/or function.

## Figures and Tables

**Table 1:** Yeast strains and genotypes.

Host	Strain	Genotype	Feature	References
*P. pastoris*	KM71H	*aox1::ARG4, arg4*	Strain with Mut^S^ phenotype	[58]
*P. pastoris*	SMD1168H	*pep4*	Strain without protease A activity	[59]
*S. cerevisiae*	PAP1500	*MATα ura3-52 trp1::GAL10-GAL4 lys2-801 leu2Δ1 his3Δ200pep4::HIS3prb1Δ1.6Rcan1GAL*	Overexpression of the Gal4 transcription factor	[60]
*S. cerevisiae*	AB11c	*ena1-4Δnhx1Δnha1Δ*	Deletion of endogenous cation/proton antiporters and pumps	[61]
*S. cerevisiae*	MSY6210	*MAT α leu2-3,112 ura3-52 his3200 trp1-901lys2-801suc2-9 smf1::HIS3,smf2::KANR*	Deletion of Mg^2+^ transporters	[62]
*S. cerevisiae*	MSY6211	*MAT a leu2-3,112 ura3-52 his3200 trp1-901 ade2-101 suc2-9 smf3::LEU2*	Deletion of Mg^2+^ transporters	[62]
*P. pastoris*	GS115	*his4*	Deletion of histidinol dehydrogenase	[63]
*S. cerevisiae*	EBY.S7	*MATα hxt1-17Δgal2Δagt1Δstl1Δleu2-3,112 ura3-52 trp1-289 his3-Δ1 MAL2–8c SUC2 hxtΔfgy1*	Deletion of hexose transporters	[64]
*S. cerevisiae*	EBY.F4–1	*MATα hxt1-17Δgal2Δagt1Δstl1Δleu2-3,112 ura3-52 trp1-289 his3-Δ1 MAL2–8c SUC2 hxtΔfgy1 fgy41*	Deletion of hexose transporters	[64]
*S. cerevisiae*	EBY.VW4000	*MATa leu2-3,112 ura3-52 trp1-289 his3-1 MAL2-8c SUC2 Δhxt1-17 Δgal2 Δstl1::loxP Δagt1::loxP Δmph2::loxP Δmph3::loxP*	Deletion of hexose transporters	[65]
*S. cerevisiae*	SDY.022	*MATa leu2-3,112 ura3-52 trp1-289 his3-∆1 MAL2-8C SUC2 ∆hxt1-17 ∆gal2 ∆agt1 ∆stl1 fgy1-1 erg4::kanMX*	Deletion of hexose transporters	[66]
*S. cerevisiae*	BJ5457	*MATα ura3-52 trp1 lys2-801 leu2-Δ1 his3-Δ200 pep4:HIS3 prb1-delta1.6R can1 GAL*	Protease deficient	[67]
*S. cerevisiae*	RE700A	*MATa hxt1::HIS3::hxt4 hxt5::LEU2 hxt2::HIS3hxt3::LEU2::hxt6 hxt7::HIS3*	Deletion of hexose transporters	[68]
*S. cerevisiae*	BY4742	*MATα his3Δ1 leu2Δ0 lys2Δ0 ura3Δ0*	Minimize homologous recombination	[59]
*S. cerevisiae*	BY4742 GEV	*MATa, (PGAL10+gal1)Δ::loxP, leu2Δ0::PACT1-GEV-NatMX, gal4Δ::LEU2, HAP1+*	Minimize homologous recombination	[69]
*P. pastoris*	SMD1168H	*pep4*	Protease A deficiency	[59]
*S. cerevisiae*	FAB158	*MATa his3- Δ200 leu2- Δ1 lys2-801 trp1- Δ1 ade2-101 ura3-52 tat2 Δ::HIS3*	Deletion of tryptophan transporter	[70]
*S. cerevisiae*	TMY203	*MATa his3- Δ200 leu2- Δ1 lys2-801 trp1- Δ1 ade2-101 ura3-52 tat1 Δ::kanMX4 tat2Δ::LEU2*	Deletion of tryptophan transporters	[70]
*S. cerevisiae*	FAY18A	*MATa his3- Δ200 leu2- Δ1 lys2-801 trp1- Δ1 ade2-101 ura3-52 HPG1-1, Rsp5^P514T^*	Deletion of Rsp5 ubiquitin ligase	[70]
*S. cerevisiae*	XPY1263a	*MATa thi3Δ::natMX thi7D::kanMX*	Deletion of thiamine transporter	[71]
*S. cerevisiae*	BY4741mp	*MATa; his3Δ1; leu2Δ0; met15Δ0; ura3Δ0; mir1Δ; pic2Δ*	Deletion of phosphate and copper transporter	[72]
*S. cerevisiae*	BY4741 pic2Δ	*MATa, leu2,met15, ura3, his3, PIC2::KANMX*	Deletion of copper transporter	[73]
*S. cerevisiae*	WB-12	*MATα ade2-1 trp1-1 ura3-1 can1-100 aac1::LEU2 aac2::HIS3*	Deletion of adenine nucleotide carriers	[74]
*S. cerevisiae*	JL1-3Δ2	*Matα leu2-3,112 his3-11,15 ade2-1 trp1-1 ura3-1can1-100 anc1::LEU2 Δ anc2::HIS3 anc3::URA3*	Deletion of adenine nucleotide carriers	[75]
*S. cerevisiae*	W303-B1	*Mata; ade2-1; his3-11, -15; leu2-3, -112; ura3-1; can^R^; cyr^+^*	Poor leucine uptake	[76]
*S. cerevisiae*	W303-1A	*MATa: ade2-2; trp1-1; can1-100; leu2-3, 112; his 3-11, 15; ura3-1*	Poor leucine uptake	[77]
*S. cerevisiae*	W303	*MATa/MATα (leu2-3,112 trp1-1 can1-100 ura3-1 ade2-1 his3-11,15) [phi^+^]*	Poor leucine uptake	[78]
*S. cerevisiae*	W303 Wagc1*Δ*	*Mat a/Mat a, ura3-1/ura3-1, trp1- Δ2/trp1- Δ2, leu2-3,112/leu2-3,112, his3-11/his3-11, ade2-1/ade2-1, can1-100/can1-100*	Poor leucine uptake; deletion of AGC1 carrier	[79]
*S. cerevisiae*	PW001	*BY4741 agc1∆::URA3*	Deletion of AGC1 carrier	[80]
*S. cerevisiae*	PW002	*BY4741 agc1∆::HIS3*	Deletion of AGC1 carrier	[80]
*S. cerevisiae*	*ΔArg11* Y02386	*MATa; his3Δ1; leu2Δ0; met15Δ0; ura3D0; YOR130c::KanMX4*	Deletion of ornithine transporter 1	[81]
*S. cerevisiae*	*ΔAnt1* BJ1991	*MATa, leu2, trp1, ura3-251, prb1-1122, and pep4-3*	Deletion of ANT1 carrier	[82]
*S. cerevisiae*	TCY119	*MAT α ura3–52 leu2–3, 112 trp1-Δ1 ade2 his3-Δ1::hisG aac1-Δ1::hisG aac2-Δ1::kanMX6 aac3-Δ1::hisG [r+, TRP1]*	Deletion of AAC1, AAC2 and AAC3 carriers	[83]
*S. cerevisiae*	W303	*his3-11,15; ade2-1; leu2-3,112; ura3-1; trp1-1; can1-100; RIM2/RIM2::kanMX*	Poor leucine uptake; deletion of pyrimidine nucleotide carrier	[84]
*S. cerevisiae*	ST9352	*MATa, aro10Δ, pdc5Δ, pTEF1->ARO7, pPGK1->ARO4, pTEF1->FjTAL, pTEF1->HsSLC25A44*	Alteration of aromatic amino acid metabolism	[85]
*S. cerevisiae*	CEN.PK113-7D *ndt1Δndt2Δ*	*MATa MAL2-8c SUC2 ndt1Δndt2Δ*	Deletion of NAD transporter	[86]
*S. cerevisiae*	BY4742 *ndt1Δndt2Δ*	*MATα his3Δ1 leu2Δ0 lys2Δ0 ura3Δ0, ndt1Δndt2Δ*	Deletion of NAD transporter	[87]
*S. cerevisiae*	FOH33	*MATα MAL2-8^c^ SUC2 his3Δ ura3-52 hfd1Δ pox1Δ faa1Δ faa4Δ adh6Δ::kanMX gal80Δ gal1/10/7Δ::(GAL7p-MmCAR-ADH1t)+(GAL3p-npgA-FBA1t); pAOH9*	Deletion of alcohol dehydrogenase	[88]
*S. cerevisiae*	KY114	*MATα, gal, ura3-52, trp1, lys2, ade2, his d2000*	Deletion of thymidine transport	[89]
*S. cerevisiae*	BY4742-YBR021W	*MATα, his3, leu2, lys2, ura3, ΔFUR4*	Deletion of uridine permease	[90]
*S. cerevisiae*	DY150	*Mata ade2-1 his3-11 leu2-3,112 trp1-1 ura3-52 can1-100(oc)*	Deletion of Fe^2+^/Mn^2+^ transporter	[91]
*S. cerevisiae*	YPL1	*MATα fui1 Δ::* *HIS3, ura3-52, lys2-801, HIS3 Δ*	Deletion of uridine permease	[92]
*S. cerevisiae*	W303-Δpep4	*leu2-3,112 trp1-1 can1-100 ura3-1 ade2-1 hwas3-11,15 Δ pep4 MATα*	Deletion of vacuolar endopeptidase Pep4	[93]
*S. cerevisiae*	W303-1A	*MATa ade2-1, can1-100, cyh2, his3-11,15, leu1-c, leu2-3,112, trp1-1, ura3-1*	No growth in absence of adenine	[94]
*S. cerevisiae*	FGY217	*MATα, ura3-52, lys2 Δ 201 and pep4 Δ*	Deletion of vacuolar endopeptidase Pep4	[95]
*S. cerevisiae*	BY4743	*Mata/α his3Δ1/ his3Δ1 leu2Δ0/ leu2Δ0 lys2Δ0/+ met15Δ0/+ ura3Δ0/ ura3Δ0*	Essential genes deletion	[96]
*S. cerevisiae*	BY4743 *Δpmr1:*:KanMX	*Mata/α his3Δ1/ his3Δ1 leu2Δ0/ leu2Δ0 lys2Δ0/+ met15Δ0/+ ura3Δ0/ ura3Δ0, Δpmr1:*:KanMX	Deletion of essential genes and Pmr1 Ca^2+^ and Mn^2+^ transporter	[97]
*S. cerevisiae*	ctr1	*MATa ura3 lys2 ade2 trp1 his3 leu2 Dctr1::LEU2*	Deletion of CTR1 transporter	[98]
*S. cerevisiae*	YPH499	*MATa ura3-52 lys2-801ade2-101 trp1-Δ63 his3-Δ200 leu2-Δ1*		[99]
*S. cerevisiae*	YPH500	*MAT α/ura3–52/lys2–801/ade2–101/trp1-Δ63/his3-Δ200/leu2-Δ1*		[100]
*S. cerevisiae*	STY50	*MATa,his4-401,leu2-3,-112,trp1-1,ura3-52,HOL1-1,suc2::LEU2*	Deletion of invertase 2	[101]
*P. pastoris*	CBS7435	Wild type		[102]
*S. cerevisiae*	YPH501	*MATa/MATα ura3-52/ura3-52 lys2-801/lys2-801 ade2-101/ade2-101 trp1-Δ63/ trp1-Δ63 his3- Δ200/his3- Δ200 Ieu2- Δ1/leu2- Δ1*		[100]
*S. cerevisiae*	CB001L	*MATα, leu2, trp1, ura3, prb^−^, pep4::LEU2*	Deletion of vacuolar proteinase A and B	[103]
*S. cerevisiae*	JRY472	*Δmpc1/2/3*	Deletion of pyruvate carriers	[104]
*S. cerevisiae*	BY4741 *gdt1Δpmr1Δ*	*MATa his3Δ1 leu2Δ0 met15Δ0 ura3Δ0 gdt1::KanMX4 pmr1::KanMX4*	Deletion of Ca^2+^ and Mg^2+^ transporters	[105]

**Table 2:** Plasmids: promoters and regulation.

Plasmid	Promoter	Type	Gene Product	Regulation	References
pPICZB	AOX	Inducible	Alcohol oxydase	Methanol (+)	[108]
pYES2	GAL1	Inducible	Galactokinase	Galactose (+)/Glucose (−)	[109]
YEp4H7	HXT7	Constitutive	hexose transporter HXT7	Low glucose (+)	[110]
p426H7	HXT7	Constitutive	hexose transporter HXT7	Low glucose (+)	[110]
pVT102	ADH1	Constitutive	Alcohol dehydrogenase I	Ethanol (+)	[111]
P426-GPD	GPD	Constitutive	Glyceraldehyde—3 -phosphate dehydrogenase	Glucose	[112,113]
pEMBLyex4	CYC-GAL	Hybrid/inducible	Cytochrome C1, GAL1-GAL10 intergenic promoter	Galactose (+)	[114]
p426MET25	MET25	Inducible	methionine and cysteine synthase	methionine (−)	[115]
YEpM	Pma1	Constitutive	Plasma membrane H^+^-ATPase		[67]
pPGK	PGK1	Constitutive	3-phosphoglycerate kinase	Glucose	[61,116]
pYES2.1	GAL1	Inducible	Galactokinase	Galactose (+)/Glucose (−)	[109]
pYES	GAL1	Inducible	Galactokinase	Galactose (+)/Glucose (−)	[109]
pPICHOLI	AOX	Inducible	Alcohol oxydase	Methanol (+)	[108]
pSM1052	PGK1	Constitutive	Phosphatidyl glycerol kinase—1	Glucose	[61,116]
pPIC3L	AOX	Inducible	Alcohol oxydase	Methanol (+)	[108]
pPIC3.5K	AOX	Inducible	Alcohol oxydase	Methanol (+)	[108]
pRS315	TDH3	Constitutive	Glyceraldehyde—3-phosphate dehydrogenase	Glucose	[112,113]
pXP951	THI7		Thiamine transporter		[71]
pCM188	CYC1	Constitutive	Cytochrome c oxidase	glucose (−)	[117]
YEp352	Lac	Inducible	Β-galactosidase	Lactose (+)	[72]
pRS314-YA2P	yAAC2	Intrinsic	Yeast ADP/ATP translocase	Glucose (−)	[74]
pRS424	yAAC2	Intrinsic	Yeast ADP/ATP translocase	Glucose (−)	[74]
pYeDP-1/8-10	GAL10-CYC1	Hybrid/inducible	Cytochrome C1, GAL10 promoter	Galactose (+)	[114]
pCGS110	GAL1	Inducible	Galactokinase	Galactose (+)/Glucose (−)	[109]
pYeDP60	GAL10-CYC1	Hybrid/inducible	Cytochrome C1, GAL10 promoter	Galactose (+)	[114]
pGal110	GAL1/GAL10	Inducible	Galactokinase/epimerase	glucose (−)/galactose (+)	[109]
pCGS110	GAL1	Inducible	Galactokinase	Galactose (+)/Glucose (−)	[109]
pYX142	TPI	Constitutive	Triose-phosphateisomerase		[118]
pEL30	CTA1	Inducible	Catalase	Oleate (+)	[119]
pESC-Leu2d	GAL1	Inducible	Galactokinase	Galactose (+)/Glucose (−)	[109]
pRS42H	RIM2		Yeast mitochondrial pyrimidine nucleotide carrier		[84]
pcfB9056	TEF	Constitutive	Translational elongation factor EF-1 α		[120]
YCplac33-NAT_MCART1	TEF	Constitutive	Translational elongation factor EF-1 α		[120]
pRS415	TEF	Constitutive	Translational elongation factor EF-1 α		[120]
pIYH01	HXT6, HXT7	Constitutive	Hexose transporter 6 and 7	Low glucose (+)	[88]
pYPGE15	PGK1	Constitutive	3-phosphoglycerate kinase	Glucose	[61,116]
pDDGFP-2	GAL1	Inducible	Galactokinase	Galactose (+)/Glucose (−)	[109]
pPICZ	AOX	Inducible	Alcohol oxydase	Methanol (+)	[108]
pYES-DEST52	GAL1	Inducible	Galactokinase	Galactose (+)/Glucose (−)	[109]
pDB20	ADH1	Constitutive	Alcohol dehydrogenase I	Ethanol (+)	[121]
pYEX-BX	CUP1	Inducible	metallothionein	Cu(II) (+)	[122]
pPIC9K	AOX	Inducible	Alcohol oxydase	Methanol (+)	[108]
pKTΔATG	GPD	Constitutive	Glyceraldehyde—3-phosphate dehydrogenase	Glucose	[113]
p426GFP	GPD	Constitutive	Glyceraldehyde—3-phosphate dehydrogenase	Glucose	[112,113]
pYEScupFLAGK or pYEScupFLAGE	CUP1	Inducible	Metallothionein	Cu(II) (+)	[122]
pYEX-BESN	CUP1	Inducible	Metallothionein	Cu(II) (+)	[122]
pBEVY-GU	GAL1	Inducible	Galactokinase	Galactose (+)/Glucose (−)	[109]
pRS416	TPI1	Constitutive	Triose-phosphate isomerase,		[105]
pPICZA	AOX	Inducible	Alcohol oxydase	Methanol (+)	[108]

**Table 3:** Purification and transport studies.

Protein/Alias	Strain Feature/Deleted Gene	Tag	System of Study	Detection Method	Findings	References
SLC1A5/ASCT2	None	C-Ter 6His	PL	R, FM	F, K, I	[30,33,195,196,197,198,199,200]
SLC2A1/GLUT1	Tolerates copper induction/*Pep4*	6His	-	WB	E	[201]
SLC2A1/GLUT1	*hxt^0^*	-	IC	C, R	L, K	[64]
SLC2A1/GLUT1	*hxt^0^*	-	IC	R	I	[202]
SLC2A1/GLUT1	-	C-Ter 8His	PL	R	P, TA	[180]
SLC2A1/GLUT1	*hxt^0^*	N-Ter 6His	IC	R	K, I	[203]
SLC2A1/GLUT1	*hxt^0^*	C-Ter HA-6His	IC	C, R	L, K, I	[68]
SLC2A1/GLUT2	*hxt^0^*	-	IC	R	I	[204]
SLC2A2/GLUT2	-	C-Ter-GFP-8His	-	FM, FSEC	L, P	[60]
SLC2A2/GLUT2	*hxt^0^*	-	IC	FM, R	L, I	[66,202]
SLC2A2/GLUT2	*hxt^0^*	-	IC	R	I	[204]
SLC2A3/GLUT3	*hxt^0^*	-	IC	R	I	[66,202]
SLC2A3/GLUT3	*hxt^0^*	-	IC	R	I	[204]
SLC2A4/GLUT4	*hxt^0^*	-	IC	C, R	L, K	[64,202]
SLC2A4/GLUT4	*hxt^0^*	-	IC	R	I	[204]
SLC2A5/GLUT5	*hxt^0^*	GFP	IC	FM, R	L, K, I	[202,205]
SLC2A5/GLUT5	*hxt^0^*	-	IC	R	I	[204]
SLC4A1/AE1	*Pep4*	-	PL	C, FM, R	L, P, F	[67]
SLC4A1/AE1	*Pep4*	N-Ter HA; Internal HA; N-ter yeGFP	IC	FM, AEC	L, F	[59]
SLC4A1/AE1	*end3*	N-Ter HA; Internal HA; N-ter yeGFP	IC	FM, EM, WB	L	[69]
SLC5A1/SGLT1	-	eGFP	PLM	FM, CM	L, P, F	[168]
SLC5A6/SMVT	-	C-Ter FLAG 6His	PL	WB, R	P, TA, K	[58]
SLC7A5/LAT1	-	N-Ter StrepTagII	-	WB	E	[206]
SLC7A5/LAT1	-	N-Ter 10His	IC	R	K, I, TA	[190,207]
SLC7A6/y+LAT2	-	N-Ter StrepTagII	ND	WB	E	[206]
SLC7A8/LAT2	-	N-Ter StrepTagII	IC	WB, R	P, TA, I, K	[190,207,208]
SLC9A1/NHE1	-	C-Ter yeGFP	IC, N	FM, IMAC, CD	L, P	[208]
SLC9B2/NHA2	*ena1-4, nhx1, nha1*	N-Ter 9His, or N-Ter GFP	IC	SM	TA, I	[61]
SLC11A1/Nramp1	*smf1, smf2, smf3, bsd2, rer1*	C-Ter HA or GFP	IC	FM, PG	L, TI	[62]
SLC15A1/PEPT1	*his4*	-	IC	R	K	[63]
SLC15A1/PEPT1	*his4*	C-Ter Myc, 6His	IC	RFT	F, K, I	[209]
SLC15A1/PEPT1	-	-	IC	R	F, K, I	[210]
SLC15A2/PEPT2	-	-	IC	RFT	F, K, I	[211]
SLC16A10/MCT10	*Tat2*	N-ter 3HA, C-Ter GFP	IC	FM, R	L, TA	[99]
SLC19A3/Thiamine transporter 2	*thi3, thi7*	-	IC	PG, R	F, I	[71]
SLC22B1/SV2A	*hxt^0^*	-	IC	PG, R	F, K, I	[212]
SLC25A3/phosphate carrier	*mir1, pic2*	-	-	PG	CG	[72]
SLC25A3/phosphate carrier	*pic2*	-	M	SM	TA	[73]
SLC25A4/ANT1	*aac_s_*	-	M	R	TA, I	[74,213]
SLC25A4/ANT1	*aac_s_*	-	M	SM, R	K, I	[75,213]
SLC25A5/ANT2	*aac_s_*	-	M	SM, R	K,I	[75,213]
SLC25A6/ANT3	*aac_s_*	-	M	SM, R	K, I	[75,213]
SLC25A7/UCP1	-	-	M	RA	MR	[214]
SLC25A8/UCP2	-	-	PL	FM	I, A	[215]
SLC25A9/UCP3L	-	-	IC	FM, O	MP, MR	[76]
SLC25A9/UCP3L	-	-	IC	FC, XTT	MP, MR	[77]
SLC25A9/UCP3(L,S)	-	-	M	FM, EA	MP, OP	[216]
SLC25A9/UCP3	-	-	PL	FM	I, A	[215]
SLC25A12/Aralar1	*agc1*	-	IC	SM	TI	[79]
SLC25A13/Citrin	*agc1*	-	IC	SM	TI	[79]
SLC25A13/Citrin	*agc1*	C-Ter GFP	IC	F	L	[80]
SLC25A15/ORNT1	*Arg11*	C-Ter V5, His	IC	PG	CG	[81]
SLC25A16/ORNT2	*Arg11*	C-Ter V5, His	IC	PG	CG	[81]
SLC25A17/PMP34	*Ant1*	C-Ter 6His	PL	IA, R	L, TA	[82]
SLC25A31/ANT4	*Aac_s_*	C-Ter V5	IC, M	CG, R	TA, I	[213,217]
SLC25A33/PNC1	*Rim2*	C-Ter 6His	IC	O	MR	[84]
SLC25A36	*Rim2*	C-Ter 6His	IC	O	MR	[84]
SLC25A44	-	-	IC	H	TA	[85]
SLC25A51/MCART1	*ndt1, ndt2*	-	IC	LC-MS	TI	[86]
SLC25A51/MCART1	*ndt1, ndt2*	-	M	R	TI, TA	[87]
SLC27A1/FATP1	High fatty alcohol production	-	-	GC, SA	YS	[88]
SLC28A1/CNT1	Thymidine transporter defective	-	IC	PG	CG	[89]
SLC28A1/CNT1	*fui1*	-	IC	R	I	[218,219]
SLC28A2/CNT2	*fur4, fui1*	-	IC	IA, R	TA, K, I	[90,219,220]
SLC28A3/CNT3	*fui1*	-	IC	FM, R	L, F, K	[220]
SLC28A3/CNT3	*fui1*	-	IC	CM, R	L, I, K	[90]
SLC28A3/CNT3	*fui1*	-	IC	R	I	[219,220,221]
SLC29A1/ENT1	*Pep4*	N-Ter 10His-MBP	-	IMAC	P	[93]
SLC29A1/ENT1	*fui1*	-	IC	R	TA, K, I,	[219,220,221,222]
SLC29A1/ENT1	Expression of thyimidine kinase	-	IC, PL	R	TA, I, EB	[223]
SLC29A1/ENT1	*Fui1*	N-Ter GFP	IC	R, FM	TA, K, L	[92]
SLC29A1/ENT1	-	-	IC	R	K, I	[221]
SLC29A1/ENT1	*Fui1*	-	IC	R	TA, I	[222]
SLC29A1/ENT1	*Fui1*	-	IC	R	K, I	[94]
SLC29A2/ENT2	*Pep4*	N-Ter 10His-MBP	-	IMAC	P	[93]
SLC29A2/ENT2	*Fui1*	-	IC	R	I	[219,220,221]
SLC29A2/ENT2	*Fui1*	-	IC	R	TA, I, K	[222,224]
SLC30A1/ZnT1	*Pep4*	C-Ter His, C-Ter Strep	PL	IMAC, FM	P, TA	[95]
SLC30A8/ZnT8	-	G3-tev-G3-H10-G3-FLAG	IC, PL	R	TA, F	[181]
SLC30A10/ZnT10	*Pmr1*	C-ter V5,6His	-	PG	CG	[97]
SLC31A1/CTR1	*Ctr1*	-	-	PG	CG	[98]
SLC35A1/CST1	*-*	-	V	R	TA, I	[225]
SLC35A1/CST1	*Pep4*	GFP, 8His	IC	FM, FSEC	P, L	[226]
SLC35A1/CST1	-	GFP, 8His	-	FM, FSEC	L	[178]
SLC35A2/UGT1		-	V	R	TA	[225]
SLC35A2/UGT1	-	-	V	R	TA, F, K, I	[227,228]
SLC35B1/UGTrel1	-	GFP	PL	R	F, K, I	[189]
SLC35B4	UDP-Glc NAc transporter	N-Ter GFP	V	FM, R	L, TA	[229]
SLC35D1/ UGTrel7	*Pep4*	C-Ter 8His, C-Ter HA	V, PL	R	TA, K	[103,230]
SLC35D2/UGTrel8	-	C-Ter HA	V	FM, R	L, TA	[193]
SLC39A1/ZIP1	*Pep4*	C-Ter TEV-GFP-His	-	IMAC, FSEC	P	[231]
SLC39A2/ZIP2	*Pep4*	C-Ter TEV-GFP-His	-	FSEC	P	[231]
SLC39A11/ZIP11	*Pep4*	C-Ter TEV-GFP-His	-	-	E	[231]
SLC39A13/ZIP13	*Pep4*	C-Ter TEV-GFP-His	-	FSEC	P	[231]
SLC54A1/MPC1	*mpc1, mpc2, mpc3*	C-Ter 8His	M, PL	FM, IMAC, R, EA	L, P, TA	[104]
SLC54A2/MPC2	*mpc1, mpc2, mpc3*	C-Ter GFP	M, PL	IMAC, FM, R, EA, O	P, L, TA, MR	[104]
SLC64/TMEM165	*Gdt1p, Pmr1*	-	-	PG	CG	[105]
STRA6	None	C-Ter GFP	IC	FM, FSEC	L, P	[232]

A: activators; AEC: anion exchange chromatography; C: centrifugation; CD: circular dichroism; CG: cell growth; CM: confocal microscopy; E: protein expression; EA: enzyme activity; EB: equilibrium binding; EM: electron microscopy; F: Functional characterization; FC: flow-cytometry; FM: fluorescence measurements; GC: gas chromatography; H: HPLC analysis; I: Effect of inhibitors; IA: immunoblot analysis; IC: Intact cells; K: kinetics characterization; L: localization; M: mitochondria; MP: membrane potential measurements; MR: mitochondrial respiration; N: nanodisk; O: oxygen consumption; OP: oxidative phosphorylation; P: protein purification; PG: plate growth; PL: proteoliposomes; PLM: planar lipid membranes; R: radiolabeled compounds; RA: respiration assay; RFT: rapid filtration techniques; SM: spectroscopic measurements; TA: transport activity TI: transporter identification; V: vesicles; XTT: XTT analysis; YS: yield of substrate.

## Data Availability

Not applicable.

## References

[1] Hediger M.A., Clemencon B., Burrier R.E., Bruford E.A. (2013). The ABCs of membrane transporters in health and disease (SLC series): Introduction. Mol. Asp. Med..

[2] Zhou Y., Zhang G.Q., Wei Y.H., Zhang J.P., Zhang G.R., Ren J.X., Duan H.G., Rao Z., Wu X.A. (2013). The impact of drug transporters on adverse drug reaction. Eur. J. Drug Metab. Pharmacokinet..

[3] Yildirim M.A., Goh K.I., Cusick M.E., Barabasi A.L., Vidal M. (2007). Drug-target network. Nat. Biotechnol..

[4] Santos R., Ursu O., Gaulton A., Bento A.P., Donadi R.S., Bologa C.G., Karlsson A., Al-Lazikani B., Hersey A., Oprea T.I. (2017). A comprehensive map of molecular drug targets. Nat. Rev. Drug Discov..

[5] Scalise M., Pochini L., Giangregorio N., Tonazzi A., Indiveri C. (2013). Proteoliposomes as tool for assaying membrane transporter functions and interactions with xenobiotics. Pharmaceutics.

[6] Stein W., Litman T. (2015). Channels, Carriers, and Pumps: An Introduction to Membrane Transport.

[7] Pizzagalli M.D., Bensimon A., Superti-Furga G. (2021). A guide to plasma membrane solute carrier proteins. FEBS J..

[8] Povey S., Lovering R., Bruford E., Wright M., Lush M., Wain H. (2001). The HUGO Gene Nomenclature Committee (HGNC). Hum. Genet..

[9] Fredriksson R., Nordstrom K.J., Stephansson O., Hagglund M.G., Schioth H.B. (2008). The solute carrier (SLC) complement of the human genome: Phylogenetic classification reveals four major families. FEBS Lett..

[10] Colas C., Ung P.M., Schlessinger A. (2016). SLC Transporters: Structure, Function, and Drug Discovery. Medchemcomm.

[11] Forrest L.R., Zhang Y.W., Jacobs M.T., Gesmonde J., Xie L., Honig B.H., Rudnick G. (2008). Mechanism for alternating access in neurotransmitter transporters. Proc. Natl. Acad. Sci. USA.

[12] Bai X., Moraes T.F., Reithmeier R.A.F. (2017). Structural biology of solute carrier (SLC) membrane transport proteins. Mol. Membr. Biol..

[13] Cesar-Razquin A., Snijder B., Frappier-Brinton T., Isserlin R., Gyimesi G., Bai X., Reithmeier R.A., Hepworth D., Hediger M.A., Edwards A.M. (2015). A Call for Systematic Research on Solute Carriers. Cell.

[14] Junge F., Schneider B., Reckel S., Schwarz D., Dotsch V., Bernhard F. (2008). Large-scale production of functional membrane proteins. Cell Mol. Life Sci..

[15] Koepsell H. (2013). The SLC22 family with transporters of organic cations, anions and zwitterions. Mol. Asp. Med..

[16] Mueckler M., Thorens B. (2013). The SLC2 (GLUT) family of membrane transporters. Mol. Asp. Med..

[17] Stockbridge R.B., Kolmakova-Partensky L., Shane T., Koide A., Koide S., Miller C., Newstead S. (2015). Crystal structures of a double-barrelled fluoride ion channel. Nature.

[18] Thangaratnarajah C., Ruprecht J.J., Kunji E.R. (2014). Calcium-induced conformational changes of the regulatory domain of human mitochondrial aspartate/glutamate carriers. Nat. Commun..

[19] Kapoor K., Finer-Moore J.S., Pedersen B.P., Caboni L., Waight A., Hillig R.C., Bringmann P., Heisler I., Muller T., Siebeneicher H. (2016). Mechanism of inhibition of human glucose transporter GLUT1 is conserved between cytochalasin B and phenylalanine amides. Proc. Natl. Acad. Sci. USA.

[20] Garaeva A.A., Oostergetel G.T., Gati C., Guskov A., Paulino C., Slotboom D.J. (2018). Cryo-EM structure of the human neutral amino acid transporter ASCT2. Nat. Struct. Mol. Biol..

[21] Deng D., Sun P., Yan C., Ke M., Jiang X., Xiong L., Ren W., Hirata K., Yamamoto M., Fan S. (2015). Molecular basis of ligand recognition and transport by glucose transporters. Nature.

[22] Yan R., Zhao X., Lei J., Zhou Q. (2019). Structure of the human LAT1-4F2hc heteromeric amino acid transporter complex. Nature.

[23] Galluccio M., Console L., Pochini L., Scalise M., Giangregorio N., Indiveri C. (2022). Strategies for Successful Over-Expression of Human Membrane Transport Systems Using Bacterial Hosts: Future Perspectives. Int. J. Mol. Sci.

[24] Geertsma E.R., Groeneveld M., Slotboom D.J., Poolman B. (2008). Quality control of overexpressed membrane proteins. Proc. Natl. Acad. Sci. USA.

[25] Schlegel S., Hjelm A., Baumgarten T., Vikstrom D., de Gier J.W. (2014). Bacterial-based membrane protein production. Biochim. Biophys. Acta.

[26] Darby R.A., Cartwright S.P., Dilworth M.V., Bill R.M. (2012). Which yeast species shall I choose? Saccharomyces cerevisiae versus Pichia pastoris (review). Methods Mol. Biol..

[27] Byrne B. (2015). Pichia pastoris as an expression host for membrane protein structural biology. Curr. Opin. Struct. Biol..

[28] Lee J.Y., Chen H., Liu A., Alba B.M., Lim A.C. (2017). Auto-induction of Pichia pastoris AOX1 promoter for membrane protein expression. Protein Expr. Purif..

[29] Souabni H., Ezzine A., Bizouarn T., Baciou L. (2017). Functional Assembly of Soluble and Membrane Recombinant Proteins of Mammalian NADPH Oxidase Complex. Methods Mol. Biol..

[30] Pingitore P., Pochini L., Scalise M., Galluccio M., Hedfalk K., Indiveri C. (2013). Large scale production of the active human ASCT2 (SLC1A5) transporter in Pichia pastoris—Functional and kinetic asymmetry revealed in proteoliposomes. Biochim. Biophys. Acta.

[31] Claes K., Guerfal M., Callewaert N. (2015). Membrane protein expression and analysis in yeast. Methods Enzymol..

[32] Hartmann L., Kugler V., Wagner R. (2016). Expression of Eukaryotic Membrane Proteins in Pichia pastoris. Methods Mol. Biol..

[33] Scalise M., Pappacoda G., Mazza T., Console L., Pochini L., Indiveri C. (2022). Cysteine 467 of the ASCT2 Amino Acid Transporter Is a Molecular Determinant of the Antiport Mechanism. Int. J. Mol. Sci..

[34] Pedersen B.P., Kumar H., Waight A.B., Risenmay A.J., Roe-Zurz Z., Chau B.H., Schlessinger A., Bonomi M., Harries W., Sali A. (2013). Crystal structure of a eukaryotic phosphate transporter. Nature.

[35] Cregg J.M., Cereghino J.L., Shi J., Higgins D.R. (2000). Recombinant protein expression in Pichia pastoris. Mol. Biotechnol..

[36] Bill R.M. (2001). Yeast—A panacea for the structure-function analysis of membrane proteins?. Curr. Genet..

[37] Karathia H., Vilaprinyo E., Sorribas A., Alves R. (2011). Saccharomyces cerevisiae as a model organism: A comparative study. PLoS ONE.

[38] Eckart M.R., Bussineau C.M. (1996). Quality and authenticity of heterologous proteins synthesized in yeast. Curr. Opin. Biotechnol..

[39] Gemmill T.R., Trimble R.B. (1999). Overview of N- and O-linked oligosaccharide structures found in various yeast species. Biochim. Biophys. Acta.

[40] Chiba Y., Suzuki M., Yoshida S., Yoshida A., Ikenaga H., Takeuchi M., Jigami Y., Ichishima E. (1998). Production of human compatible high mannose-type (Man5GlcNAc2) sugar chains in Saccharomyces cerevisiae. J. Biol. Chem..

[41] Nakanishi-Shindo Y., Nakayama K., Tanaka A., Toda Y., Jigami Y. (1993). Structure of the N-linked oligosaccharides that show the complete loss of alpha-1,6-polymannose outer chain from och1, och1 mnn1, and och1 mnn1 alg3 mutants of Saccharomyces cerevisiae. J. Biol. Chem..

[42] Oka T., Jigami Y. (2006). Reconstruction of de novo pathway for synthesis of UDP-glucuronic acid and UDP-xylose from intrinsic UDP-glucose in Saccharomyces cerevisiae. FEBS J..

[43] Chigira Y., Oka T., Okajima T., Jigami Y. (2008). Engineering of a mammalian O-glycosylation pathway in the yeast Saccharomyces cerevisiae: Production of O-fucosylated epidermal growth factor domains. Glycobiology.

[44] Wach A. (1996). PCR-synthesis of marker cassettes with long flanking homology regions for gene disruptions in *S. cerevisiae*. Yeast.

[45] Longtine M.S., McKenzie A., Demarini D.J., Shah N.G., Wach A., Brachat A., Philippsen P., Pringle J.R. (1998). Additional modules for versatile and economical PCR-based gene deletion and modification in Saccharomyces cerevisiae. Yeast.

[46] Storici F., Resnick M.A. (2006). The delitto perfetto approach to in vivo site-directed mutagenesis and chromosome rearrangements with synthetic oligonucleotides in yeast. Methods Enzymol..

[47] Guldener U., Heck S., Fielder T., Beinhauer J., Hegemann J.H. (1996). A new efficient gene disruption cassette for repeated use in budding yeast. Nucleic Acids Res..

[48] Rothstein R. (1991). Targeting, disruption, replacement, and allele rescue: Integrative DNA transformation in yeast. Methods Enzymol..

[49] Nasmyth K.A., Reed S.I. (1980). Isolation of genes by complementation in yeast: Molecular cloning of a cell-cycle gene. Proc. Natl. Acad. Sci. USA.

[50] Chan C.S., Tye B.K. (1980). Autonomously replicating sequences in Saccharomyces cerevisiae. Proc. Natl. Acad. Sci. USA.

[51] Clarke L., Carbon J. (1980). Isolation of a yeast centromere and construction of functional small circular chromosomes. Nature.

[52] Szostak J.W., Blackburn E.H. (1982). Cloning yeast telomeres on linear plasmid vectors. Cell.

[53] Herskowitz I. (1987). Functional inactivation of genes by dominant negative mutations. Nature.

[54] Gardner J.M., Jaspersen S.L. (2014). Manipulating the yeast genome: Deletion, mutation, and tagging by PCR. Methods Mol. Biol..

[55] Orr-Weaver T.L., Szostak J.W., Rothstein R.J. (1981). Yeast transformation: A model system for the study of recombination. Proc. Natl. Acad. Sci. USA.

[56] Oldenburg K.R., Vo K.T., Michaelis S., Paddon C. (1997). Recombination-mediated PCR-directed plasmid construction in vivo in yeast. Nucleic Acids Res..

[57] Joska T.M., Mashruwala A., Boyd J.M., Belden W.J. (2014). A universal cloning method based on yeast homologous recombination that is simple, efficient, and versatile. J. Microbiol. Methods.

[58] Zehnpfennig B., Wiriyasermkul P., Carlson D.A., Quick M. (2015). Interaction of alpha-Lipoic Acid with the Human Na+/Multivitamin Transporter (hSMVT). J. Biol. Chem..

[59] Sarder H.A.M., Li X., Funaya C., Cordat E., Schmitt M.J., Becker B. (2020). Saccharomyces cerevisiae: First Steps to a Suitable Model System To Study the Function and Intracellular Transport of Human Kidney Anion Exchanger 1. mSphere.

[60] Scharff-Poulsen P., Pedersen P.A. (2013). Saccharomyces cerevisiae-based platform for rapid production and evaluation of eukaryotic nutrient transporters and transceptors for biochemical studies and crystallography. PLoS ONE.

[61] Xiang M., Feng M., Muend S., Rao R. (2007). A human Na^+^/H^+^ antiporter sharing evolutionary origins with bacterial NhaA may be a candidate gene for essential hypertension. Proc. Natl. Acad. Sci. USA.

[62] Techau M.E., Valdez-Taubas J., Popoff J.F., Francis R., Seaman M., Blackwell J.M. (2007). Evolution of differences in transport function in Slc11a family members. J. Biol. Chem..

[63] Doring F., Walter J., Will J., Focking M., Boll M., Amasheh S., Clauss W., Daniel H. (1998). Delta-aminolevulinic acid transport by intestinal and renal peptide transporters and its physiological and clinical implications. J. Clin. Investig..

[64] Wieczorke R., Dlugai S., Krampe S., Boles E. (2003). Characterisation of mammalian GLUT glucose transporters in a heterologous yeast expression system. Cell Physiol. Biochem..

[65] Wieczorke R., Krampe S., Weierstall T., Freidel K., Hollenberg C.P., Boles E. (1999). Concurrent knock-out of at least 20 transporter genes is required to block uptake of hexoses in Saccharomyces cerevisiae. Febs. Lett..

[66] Schmidl S., Tamayo Rojas S.A., Iancu C.V., Choe J.Y., Oreb M. (2020). Functional Expression of the Human Glucose Transporters GLUT2 and GLUT3 in Yeast Offers Novel Screening Systems for GLUT-Targeting Drugs. Front. Mol. Biosci..

[67] Bonar P., Casey J.R. (2010). Purification of functional human Cl^−^/HCO_3_^−^ exchanger, AE1, over-expressed in Saccharomyces cerevisiae. Protein Expr. Purif..

[68] Levine K.B., Robichaud T.K., Hamill S., Sultzman L.A., Carruthers A. (2005). Properties of the human erythrocyte glucose transport protein are determined by cellular context. Biochemistry.

[69] Li X., Cordat E., Schmitt M.J., Becker B. (2021). Boosting endoplasmic reticulum folding capacity reduces unfolded protein response activation and intracellular accumulation of human kidney anion exchanger 1 in Saccharomyces cerevisiae. Yeast.

[70] Abe F., Iida H. (2003). Pressure-induced differential regulation of the two tryptophan permeases Tat1 and Tat2 by ubiquitin ligase Rsp5 and its binding proteins, Bul1 and Bul2. Mol. Cell Biol.

[71] Huang Z., Srinivasan S., Zhang J., Chen K., Li Y., Li W., Quiocho F.A., Pan X. (2012). Discovering thiamine transporters as targets of chloroquine using a novel functional genomics strategy. PLoS Genet..

[72] Mayr J.A., Merkel O., Kohlwein S.D., Gebhardt B.R., Bohles H., Fotschl U., Koch J., Jaksch M., Lochmuller H., Horvath R. (2007). Mitochondrial phosphate-carrier deficiency: A novel disorder of oxidative phosphorylation. Am. J. Hum. Genet..

[73] Boulet A., Vest K.E., Maynard M.K., Gammon M.G., Russell A.C., Mathews A.T., Cole S.E., Zhu X., Phillips C.B., Kwong J.Q. (2018). The mammalian phosphate carrier SLC25A3 is a mitochondrial copper transporter required for cytochrome c oxidase biogenesis. J. Biol. Chem..

[74] Hatanaka T., Takemoto Y., Hashimoto M., Majima E., Shinohara Y., Terada H. (2001). Significant expression of functional human type 1 mitochondrial ADP/ATP carrier in yeast mitochondria. Biol. Pharm. Bull..

[75] De Marcos Lousa C., Trezeguet V., Dianoux A.C., Brandolin G., Lauquin G.J. (2002). The human mitochondrial ADP/ATP carriers: Kinetic properties and biogenesis of wild-type and mutant proteins in the yeast S. cerevisiae. Biochemistry.

[76] Hinz W., Faller B., Gruninger S., Gazzotti P., Chiesi M. (1999). Recombinant human uncoupling protein-3 increases thermogenesis in yeast cells. FEBS Lett..

[77] Brown A.M., Dolan J.W., Willi S.M., Garvey W.T., Argyropoulos G. (1999). Endogenous mutations in human uncoupling protein 3 alter its functional properties. FEBS Lett..

[78] Cohen R., Engelberg D. (2007). Commonly used Saccharomyces cerevisiae strains (e.g., BY4741, W303) are growth sensitive on synthetic complete medium due to poor leucine uptake. FEMS Microbiol. Lett..

[79] Cavero S., Vozza A., del Arco A., Palmieri L., Villa A., Blanco E., Runswick M.J., Walker J.E., Cerdan S., Palmieri F. (2003). Identification and metabolic role of the mitochondrial aspartate-glutamate transporter in Saccharomyces cerevisiae. Mol. Microbiol..

[80] Wongkittichote P., Tungpradabkul S., Wattanasirichaigoon D., Jensen L.T. (2013). Prediction of the functional effect of novel SLC25A13 variants using a S. cerevisiae model of AGC2 deficiency. J. Inherit. Metab. Dis..

[81] Doimo M., Lopreiato R., Basso V., Bortolotto R., Tessa A., Santorelli F.M., Trevisson E., Salviati L. (2016). Heterologous Expression in Yeast of Human Ornithine Carriers ORNT1 and ORNT2 and of ORNT1 Alleles Implicated in HHH Syndrome in Humans. JIMD Rep..

[82] Visser W.F., van Roermund C.W., Waterham H.R., Wanders R.J. (2002). Identification of human PMP34 as a peroxisomal ATP transporter. Biochem. Biophys. Res. Commun..

[83] Smith C.P., Thorsness P.E. (2008). The molecular basis for relative physiological functionality of the ADP/ATP carrier isoforms in Saccharomyces cerevisiae. Genetics.

[84] Di Noia M.A., Todisco S., Cirigliano A., Rinaldi T., Agrimi G., Iacobazzi V., Palmieri F. (2014). The human SLC25A33 and SLC25A36 genes of solute carrier family 25 encode two mitochondrial pyrimidine nucleotide transporters. J. Biol. Chem..

[85] Darbani B. (2021). Genome Evolutionary Dynamics Meets Functional Genomics: A Case Story on the Identification of SLC25A44. Int. J. Mol. Sci..

[86] Kory N., Uit de Bos J., van der Rijt S., Jankovic N., Gura M., Arp N., Pena I.A., Prakash G., Chan S.H., Kunchok T. (2020). MCART1/SLC25A51 is required for mitochondrial NAD transport. Sci. Adv..

[87] Luongo T.S., Eller J.M., Lu M.J., Niere M., Raith F., Perry C., Bornstein M.R., Oliphint P., Wang L., McReynolds M.R. (2020). SLC25A51 is a mammalian mitochondrial NAD(+) transporter. Nature.

[88] Hu Y., Zhu Z., Nielsen J., Siewers V. (2018). Heterologous transporter expression for improved fatty alcohol secretion in yeast. Metab. Eng..

[89] Vickers M.F., Young J.D., Baldwin S.A., Ellison M.J., Cass C.E. (2001). Functional production of mammalian concentrative nucleoside transporters in Saccharomyces cerevisiae. Mol. Membr. Biol..

[90] Zhang J., Smith K.M., Tackaberry T., Visser F., Robins M.J., Nielsen L.P., Nowak I., Karpinski E., Baldwin S.A., Young J.D. (2005). Uridine binding and transportability determinants of human concentrative nucleoside transporters. Mol. Pharmacol..

[91] Lin H., Kumanovics A., Nelson J.M., Warner D.E., Ward D.M., Kaplan J. (2008). A single amino acid change in the yeast vacuolar metal transporters ZRC1 and COT1 alters their substrate specificity. J. Biol. Chem..

[92] SenGupta D.J., Lum P.Y., Lai Y., Shubochkina E., Bakken A.H., Schneider G., Unadkat J.D. (2002). A single glycine mutation in the equilibrative nucleoside transporter gene, hENT1, alters nucleoside transport activity and sensitivity to nitrobenzylthioinosine. Biochemistry.

[93] Boswell-Casteel R.C., Johnson J.M., Roe-Žurž Z., Duggan K.D., Schmitz H., Hays F.A. (2018). Expression and purification of human and Saccharomyces cerevisiae equilibrative nucleoside transporters. Protein Expr. Purif..

[94] Endres C.J., Sengupta D.J., Unadkat J.D. (2004). Mutation of leucine-92 selectively reduces the apparent affinity of inosine, guanosine, NBMPR [S6-(4-nitrobenzyl)-mercaptopurine riboside] and dilazep for the human equilibrative nucleoside transporter, hENT1. Biochem. J..

[95] Cotrim C.A., Jarrott R.J., Whitten A.E., Choudhury H.G., Drew D., Martin J.L. (2021). Heterologous Expression and Biochemical Characterization of the Human Zinc Transporter 1 (ZnT1) and Its Soluble C-Terminal Domain. Front. Chem..

[96] Winzeler E.A., Shoemaker D.D., Astromoff A., Liang H., Anderson K., Andre B., Bangham R., Benito R., Boeke J.D., Bussey H. (1999). Functional characterization of the S. cerevisiae genome by gene deletion and parallel analysis. Science.

[97] Tuschl K., Clayton P.T., Gospe S.M., Gulab S., Ibrahim S., Singhi P., Aulakh R., Ribeiro R.T., Barsottini O.G., Zaki M.S. (2012). Syndrome of hepatic cirrhosis, dystonia, polycythemia, and hypermanganesemia caused by mutations in SLC30A10, a manganese transporter in man. Am. J. Hum. Genet..

[98] Zhou B., Gitschier J. (1997). hCTR1: A human gene for copper uptake identified by complementation in yeast. Proc. Natl. Acad. Sci. USA.

[99] Uemura S., Mochizuki T., Kurosaka G., Hashimoto T., Masukawa Y., Abe F. (2017). Functional analysis of human aromatic amino acid transporter MCT10/TAT1 using the yeast Saccharomyces cerevisiae. Biochim. Biophys. Acta Biomembr..

[100] Sikorski R.S., Hieter P. (1989). A system of shuttle vectors and yeast host strains designed for efficient manipulation of DNA in Saccharomyces cerevisiae. Genetics.

[101] Strahl-Bolsinger S., Scheinost A. (1999). Transmembrane topology of pmt1p, a member of an evolutionarily conserved family of protein O-mannosyltransferases. J. Biol. Chem..

[102] Kuberl A., Schneider J., Thallinger G.G., Anderl I., Wibberg D., Hajek T., Jaenicke S., Brinkrolf K., Goesmann A., Szczepanowski R. (2011). High-quality genome sequence of Pichia pastoris CBS7435. J. Biotechnol..

[103] Muraoka M., Miki T., Ishida N., Hara T., Kawakita M. (2007). Variety of nucleotide sugar transporters with respect to the interaction with nucleoside mono- and diphosphates. J. Biol. Chem..

[104] Nagampalli R.S.K., Quesnay J.E.N., Adamoski D., Islam Z., Birch J., Sebinelli H.G., Girard R., Ascencao C.F.R., Fala A.M., Pauletti B.A. (2018). Human mitochondrial pyruvate carrier 2 as an autonomous membrane transporter. Sci. Rep..

[105] Stribny J., Thines L., Deschamps A., Goffin P., Morsomme P. (2020). The human Golgi protein TMEM165 transports calcium and manganese in yeast and bacterial cells. J. Biol. Chem..

[106] Lundblad V. (2001). Yeast cloning vectors and genes. Curr. Protoc. Mol. Biol..

[107] Peng B., Williams T.C., Henry M., Nielsen L.K., Vickers C.E. (2015). Controlling heterologous gene expression in yeast cell factories on different carbon substrates and across the diauxic shift: A comparison of yeast promoter activities. Microb. Cell Fact..

[108] Ellis S.B., Brust P.F., Koutz P.J., Waters A.F., Harpold M.M., Gingeras T.R. (1985). Isolation of alcohol oxidase and two other methanol regulatable genes from the yeast Pichia pastoris. Mol. Cell Biol..

[109] West R.W., Yocum R.R., Ptashne M. (1984). Saccharomyces cerevisiae GAL1-GAL10 divergent promoter region: Location and function of the upstream activating sequence UASG. Mol. Cell Biol..

[110] Ye L., Berden J.A., van Dam K., Kruckeberg A.L. (2001). Expression and activity of the Hxt7 high-affinity hexose transporter of Saccharomyces cerevisiae. Yeast.

[111] Ruohonen L., Aalto M.K., Keranen S. (1995). Modifications to the ADH1 promoter of Saccharomyces cerevisiae for efficient production of heterologous proteins. J. Biotechnol..

[112] Musti A.M., Zehner Z., Bostian K.A., Paterson B.M., Kramer R.A. (1983). Transcriptional mapping of two yeast genes coding for glyceraldehyde 3-phosphate dehydrogenase isolated by sequence homology with the chicken gene. Gene.

[113] Bitter G.A., Egan K.M. (1984). Expression of heterologous genes in Saccharomyces cerevisiae from vectors utilizing the glyceraldehyde-3-phosphate dehydrogenase gene promoter. Gene.

[114] Pedersen P.A., Rasmussen J.H., Joorgensen P.L. (1996). Expression in high yield of pig alpha 1 beta 1 Na,K-ATPase and inactive mutants D369N and D807N in Saccharomyces cerevisiae. J. Biol. Chem..

[115] Mumberg D., Muller R., Funk M. (1994). Regulatable promoters of Saccharomyces cerevisiae: Comparison of transcriptional activity and their use for heterologous expression. Nucleic Acids Res..

[116] Tuite M.F., Dobson M.J., Roberts N.A., King R.M., Burke D.C., Kingsman S.M., Kingsman A.J. (1982). Regulated high efficiency expression of human interferon-alpha in Saccharomyces cerevisiae. EMBO J..

[117] Guarente L., Lalonde B., Gifford P., Alani E. (1984). Distinctly regulated tandem upstream activation sites mediate catabolite repression of the CYC1 gene of S. cerevisiae. Cell.

[118] Humphries A., Ationu A., Wild B., Layton D.M. (1999). The consequence of nucleotide substitutions in the triosephosphate isomerase (TPI) gene promoter. Blood Cells Mol. Dis..

[119] Elgersma Y., van den Berg M., Tabak H.F., Distel B. (1993). An efficient positive selection procedure for the isolation of peroxisomal import and peroxisome assembly mutants of Saccharomyces cerevisiae. Genetics.

[120] Steiner S., Philippsen P. (1994). Sequence and promoter analysis of the highly expressed TEF gene of the filamentous fungus Ashbya gossypii. Mol. Gen. Genet..

[121] Becker D.M., Fikes J.D., Guarente L. (1991). A cDNA encoding a human CCAAT-binding protein cloned by functional complementation in yeast. Proc. Natl. Acad. Sci. USA.

[122] Etcheverry T. (1990). Induced expression using yeast copper metallothionein promoter. Methods Enzymol..

[123] Chen X., Li S., Liu L. (2014). Engineering redox balance through cofactor systems. Trends Biotechnol..

[124] Na D., Kim T.Y., Lee S.Y. (2010). Construction and optimization of synthetic pathways in metabolic engineering. Curr. Opin. Microbiol..

[125] Tschumper G., Carbon J. (1980). Sequence of a yeast DNA fragment containing a chromosomal replicator and the TRP1 gene. Gene.

[126] Struhl K., Davis R.W. (1980). A physical, genetic and transcriptional map of the cloned his3 gene region of Saccharomyces cerevisiae. J. Mol. Biol..

[127] Rose M., Grisafi P., Botstein D. (1984). Structure and function of the yeast URA3 gene: Expression in Escherichia coli. Gene.

[128] Brachmann C.B., Davies A., Cost G.J., Caputo E., Li J., Hieter P., Boeke J.D. (1998). Designer deletion strains derived from Saccharomyces cerevisiae S288C: A useful set of strains and plasmids for PCR-mediated gene disruption and other applications. Yeast.

[129] Britton Z., Young C., Can O., McNeely P., Naranjo A., Robinson A.S. (2011). Membrane Protein Expression in Saccharomyces cerevisiae. Production of Membrane Proteins: Strategies for Expression and Isolation.

[130] Hinnen A., Hicks J.B., Fink G.R. (1978). Transformation of yeast. Proc. Natl. Acad. Sci. USA.

[131] Armaleo D., Ye G.N., Klein T.M., Shark K.B., Sanford J.C., Johnston S.A. (1990). Biolistic nuclear transformation of Saccharomyces cerevisiae and other fungi. Curr. Genet..

[132] Costanzo M.C., Fox T.D. (1988). Transformation of yeast by agitation with glass beads. Genetics.

[133] Burgers P.M., Percival K.J. (1987). Transformation of yeast spheroplasts without cell fusion. Anal. Biochem..

[134] Gietz D., St Jean A., Woods R.A., Schiestl R.H. (1992). Improved method for high efficiency transformation of intact yeast cells. Nucleic Acids Res..

[135] Gietz R.D., Schiestl R.H., Willems A.R., Woods R.A. (1995). Studies on the transformation of intact yeast cells by the LiAc/SS-DNA/PEG procedure. Yeast.

[136] Delorme E. (1989). Transformation of Saccharomyces cerevisiae by electroporation. Appl. Environ. Microbiol..

[137] Thompson J.R., Register E., Curotto J., Kurtz M., Kelly R. (1998). An improved protocol for the preparation of yeast cells for transformation by electroporation. Yeast.

[138] Suga M., Hatakeyama T. (2003). High-efficiency electroporation by freezing intact yeast cells with addition of calcium. Curr. Genet..

[139] Kawai S., Hashimoto W., Murata K. (2010). Transformation of Saccharomyces cerevisiae and other fungi: Methods and possible underlying mechanism. Bioeng. Bugs..

[140] Ito H., Fukuda Y., Murata K., Kimura A. (1983). Transformation of intact yeast cells treated with alkali cations. J. Bacteriol..

[141] Hashimoto H., Morikawa H., Yamada Y., Kimura A. (1985). Novel method for transformation of intact yeast cells by electroinjection of plasmid DNA. Appl. Microbiol. Biotechnol..

[142] Becker D.M., Guarente L. (1991). High-efficiency transformation of yeast by electroporation. Methods Enzymol..

[143] Sherman F. (2002). Getting started with yeast. Methods Enzymol..

[144] Gasser B., Mattanovich D. (2007). Antibody production with yeasts and filamentous fungi: On the road to large scale?. Biotechnol. Lett..

[145] Hackel B.J., Huang D.G., Buboz J.C., Wang X.X., Shusta E.V. (2006). Production of soluble and active transferrin receptor-targeting single-chain antibody using Saccharomyces cerevisiae. Pharm. Res. Dordr..

[146] Joubert O., Nehme R., Bidet M., Mus-Veteau I. (2010). Heterologous Expression of Human Membrane Receptors in the Yeast Saccharomyces cerevisiae. Heterologous Expr. Membr. Proteins Methods Protoc..

[147] Ferndahl C., Bonander N., Logez C., Wagner R., Gustafsson L., Larsson C., Hedfalk K., Darby R.A., Bill R.M. (2010). Increasing cell biomass in Saccharomyces cerevisiae increases recombinant protein yield: The use of a respiratory strain as a microbial cell factory. Microb. Cell Fact..

[148] Walsh G. (2010). Biopharmaceutical benchmarks 2010. Nat. Biotechnol..

[149] Vorauer-Uhl K., Lhota G. (2019). Assessing the Quality of Recombinant Products Made in Yeast. Methods Mol. Biol..

[150] Treco D.A., Lundblad V. (2001). Preparation of yeast media. Curr. Protoc. Mol. Biol..

[151] Pronk J.T., Yde Steensma H., Van Dijken J.P. (1996). Pyruvate metabolism in Saccharomyces cerevisiae. Yeast.

[152] Rozpedowska E., Hellborg L., Ishchuk O.P., Orhan F., Galafassi S., Merico A., Woolfit M., Compagno C., Piskur J. (2011). Parallel evolution of the make-accumulate-consume strategy in Saccharomyces and Dekkera yeasts. Nat. Commun..

[153] Otterstedt K., Larsson C., Bill R.M., Stahlberg A., Boles E., Hohmann S., Gustafsson L. (2004). Switching the mode of metabolism in the yeast Saccharomyces cerevisiae. EMBO Rep..

[154] Mentel M., Chovancikova P., Zeman I., Polcic P. (2021). Learning from Yeast about Mitochondrial Carriers. Microorganisms.

[155] Monne M., Vozza A., Lasorsa F.M., Porcelli V., Palmieri F. (2019). Mitochondrial Carriers for Aspartate, Glutamate and Other Amino Acids: A Review. Int. J. Mol. Sci..

[156] Ferramosca A., Zara V. (2021). Mitochondrial Carriers and Substrates Transport Network: A Lesson from Saccharomyces cerevisiae. Int. J. Mol. Sci..

[157] Di Rosa M.C., Guarino F., Conti Nibali S., Magri A., De Pinto V. (2021). Voltage-Dependent Anion Selective Channel Isoforms in Yeast: Expression, Structure, and Functions. Front. Physiol..

[158] Bonander N., Hedfalk K., Larsson C., Mostad P., Chang C., Gustafsson L., Bill R.M. (2005). Design of improved membrane protein production experiments: Quantitation of the host response. Protein Sci..

[159] Bonander N., Darby R.A., Grgic L., Bora N., Wen J., Brogna S., Poyner D.R., O’Neill M.A., Bill R.M. (2009). Altering the ribosomal subunit ratio in yeast maximizes recombinant protein yield. Microb. Cell Fact..

[160] Bawa Z., Bland C.E., Bonander N., Bora N., Cartwright S.P., Clare M., Conner M.T., Darby R.A., Dilworth M.V., Holmes W.J. (2011). Understanding the yeast host cell response to recombinant membrane protein production. Biochem. Soc. Trans..

[161] Kurtzman C.P. (2009). Biotechnological strains of Komagataella (Pichia) pastoris are Komagataella phaffii as determined from multigene sequence analysis. J. Ind. Microbiol. Biotechnol..

[162] Cregg J.M., Vedvick T.S., Raschke W.C. (1993). Recent advances in the expression of foreign genes in Pichia pastoris. Biotechnology.

[163] Hamilton S.R., Gerngross T.U. (2007). Glycosylation engineering in yeast: The advent of fully humanized yeast. Curr. Opin. Biotechnol..

[164] Emmerstorfer-Augustin A., Wriessnegger T., Hirz M., Zellnig G., Pichler H. (2019). Membrane Protein Production in Yeast: Modification of Yeast Membranes for Human Membrane Protein Production. Methods Mol. Biol..

[165] Abad S., Kitz K., Hormann A., Schreiner U., Hartner F.S., Glieder A. (2010). Real-time PCR-based determination of gene copy numbers in Pichia pastoris. Biotechnol. J..

[166] Andre N., Cherouati N., Prual C., Steffan T., Zeder-Lutz G., Magnin T., Pattus F., Michel H., Wagner R., Reinhart C. (2006). Enhancing functional production of G protein-coupled receptors in Pichia pastoris to levels required for structural studies via a single expression screen. Protein Sci..

[167] Sunga A.J., Tolstorukov I., Cregg J.M. (2008). Posttransformational vector amplification in the yeast Pichia pastoris. FEMS Yeast Res..

[168] Suades A., Alcaraz A., Cruz E., Álvarez-Marimon E., Whitelegge J.P., Manyosa J., Cladera J., Perálvarez-Marín A. (2019). Structural biology workflow for the expression and characterization of functional human sodium glucose transporter type 1 in Pichia pastoris. Sci. Rep..

[169] Bird L.E., Nettleship J.E., Jarvinen V., Rada H., Verma A., Owens R.J. (2016). Expression Screening of Integral Membrane Proteins by Fusion to Fluorescent Reporters. Adv. Exp. Med. Biol..

[170] Brooks C.L., Morrison M., Lemieux M.J. (2013). Rapid expression screening of eukaryotic membrane proteins in Pichia pastoris. Protein Sci..

[171] Kastilan R., Boes A., Spiegel H., Voepel N., Chudobova I., Hellwig S., Buyel J.F., Reimann A., Fischer R. (2017). Improvement of a fermentation process for the production of two PfAMA1-DiCo-based malaria vaccine candidates in Pichia pastoris. Sci. Rep..

[172] Cregg J.M., Madden K.R., Barringer K.J., Thill G.P., Stillman C.A. (1989). Functional characterization of the two alcohol oxidase genes from the yeast Pichia pastoris. Mol. Cell Biol..

[173] Bawa Z., Routledge S.J., Jamshad M., Clare M., Sarkar D., Dickerson I., Ganzlin M., Poyner D.R., Bill R.M. (2014). Functional recombinant protein is present in the pre-induction phases of Pichia pastoris cultures when grown in bioreactors, but not shake-flasks. Microb. Cell Fact..

[174] Cos O., Ramon R., Montesinos J.L., Valero F. (2006). Operational strategies, monitoring and control of heterologous protein production in the methylotrophic yeast Pichia pastoris under different promoters: A review. Microb. Cell Fact..

[175] Vogl T., Sturmberger L., Fauland P.C., Hyden P., Fischer J.E., Schmid C., Thallinger G.G., Geier M., Glieder A. (2018). Methanol independent induction in Pichia pastoris by simple derepressed overexpression of single transcription factors. Biotechnol. Bioeng..

[176] Weis R., Luiten R., Skranc W., Schwab H., Wubbolts M., Glieder A. (2004). Reliable high-throughput screening with Pichia pastoris by limiting yeast cell death phenomena. FEMS Yeast Res..

[177] Routledge S.J., Mikaliunaite L., Patel A., Clare M., Cartwright S.P., Bawa Z., Wilks M.D., Low F., Hardy D., Rothnie A.J. (2016). The synthesis of recombinant membrane proteins in yeast for structural studies. Methods.

[178] Vogl T., Thallinger G.G., Zellnig G., Drew D., Cregg J.M., Glieder A., Freigassner M. (2014). Towards improved membrane protein production in Pichia pastoris: General and specific transcriptional response to membrane protein overexpression. New Biotechnol..

[179] Guyot L., Hartmann L., Mohammed-Bouteben S., Caro L., Wagner R. (2020). Preparation of Recombinant Membrane Proteins from Pichia pastoris for Molecular Investigations. Curr. Protoc. Protein Sci..

[180] Alisio A., Mueckler M. (2010). Purification and characterization of mammalian glucose transporters expressed in Pichia pastoris. Protein Expr. Purif..

[181] Daniels M.J., Jagielnicki M., Yeager M. (2020). Structure/Function Analysis of human ZnT8 (SLC30A8): A Diabetes Risk Factor and Zinc Transporter. Curr. Res. Struct. Biol..

[182] Teo A.C.K., Lee S.C., Pollock N.L., Stroud Z., Hall S., Thakker A., Pitt A.R., Dafforn T.R., Spickett C.M., Roper D.I. (2019). Analysis of SMALP co-extracted phospholipids shows distinct membrane environments for three classes of bacterial membrane protein. Sci. Rep..

[183] Parmar M., Rawson S., Scarff C.A., Goldman A., Dafforn T.R., Muench S.P., Postis V.L.G. (2018). Using a SMALP platform to determine a sub-nm single particle cryo-EM membrane protein structure. Biochim. Biophys. Acta Biomembr..

[184] Sun C., Benlekbir S., Venkatakrishnan P., Wang Y., Hong S., Hosler J., Tajkhorshid E., Rubinstein J.L., Gennis R.B. (2018). Structure of the alternative complex III in a supercomplex with cytochrome oxidase. Nature.

[185] Qiu W., Fu Z., Xu G.G., Grassucci R.A., Zhang Y., Frank J., Hendrickson W.A., Guo Y. (2018). Structure and activity of lipid bilayer within a membrane-protein transporter. Proc. Natl. Acad. Sci. USA.

[186] King M.S., Boes C., Kunji E.R. (2015). Membrane protein expression in Lactococcus lactis. Methods Enzymol..

[187] Martens C. (2020). Membrane Protein Production in Lactococcus lactis for Structural Studies. Methods Mol. Biol..

[188] Galluccio M., Mazza T., Scalise M., Sarubbi M.C., Indiveri C. (2022). Bacterial over-expression of functionally active human CT2 (SLC22A16) carnitine transporter. Mol. Biol. Rep..

[189] Schwarzbaum P.J., Schachter J., Bredeston L.M. (2022). The broad range di- and trinucleotide exchanger SLC35B1 displays asymmetrical affinities for ATP transport across the ER membrane. J. Biol. Chem..

[190] Kantipudi S., Fotiadis D. (2021). Yeast Cell-Based Transport Assay for the Functional Characterization of Human 4F2hc-LAT1 and -LAT2, and LAT1 and LAT2 Substrates and Inhibitors. Front. Mol. Biosci..

[191] Schmidt T.G., Skerra A. (2007). The Strep-tag system for one-step purification and high-affinity detection or capturing of proteins. Nat. Protoc..

[192] Kimple M.E., Brill A.L., Pasker R.L. (2013). Overview of affinity tags for protein purification. Curr. Protoc. Protein Sci..

[193] Ishida N., Kuba T., Aoki K., Miyatake S., Kawakita M., Sanai Y. (2005). Identification and characterization of human Golgi nucleotide sugar transporter SLC35D2, a novel member of the SLC35 nucleotide sugar transporter family. Genomics.

[194] Aller S.G., Unger V.M. (2006). Projection structure of the human copper transporter CTR1 at 6-A resolution reveals a compact trimer with a novel channel-like architecture. Proc. Natl. Acad. Sci. USA.

[195] Scalise M., Mazza T., Pappacoda G., Pochini L., Cosco J., Rovella F., Indiveri C. (2020). The Human SLC1A5 Neutral Amino Acid Transporter Catalyzes a pH-Dependent Glutamate/Glutamine Antiport, as Well. Front. Cell Dev. Biol..

[196] Scalise M., Pochini L., Console L., Pappacoda G., Pingitore P., Hedfalk K., Indiveri C. (2018). Cys Site-Directed Mutagenesis of the Human SLC1A5 (ASCT2) Transporter: Structure/Function Relationships and Crucial Role of Cys467 for Redox Sensing and Glutamine Transport. Int. J. Mol. Sci..

[197] Scalise M., Pochini L., Pingitore P., Hedfalk K., Indiveri C. (2015). Cysteine is not a substrate but a specific modulator of human ASCT2 (SLC1A5) transporter. FEBS Lett..

[198] Scalise M., Pochini L., Panni S., Pingitore P., Hedfalk K., Indiveri C. (2014). Transport mechanism and regulatory properties of the human amino acid transporter ASCT2 (SLC1A5). Amino Acids.

[199] Mazza T., Scalise M., Pappacoda G., Pochini L., Indiveri C. (2021). The involvement of sodium in the function of the human amino acid transporter ASCT2. FEBS Lett..

[200] Scalise M., Pochini L., Cosco J., Aloe E., Mazza T., Console L., Esposito A., Indiveri C. (2019). Interaction of Cholesterol With the Human SLC1A5 (ASCT2): Insights Into Structure/Function Relationships. Front. Mol. Biosci..

[201] Simons C.H., Weinglass A.B., Baldwin S.A. (1997). Studies on the expression of the human erythrocyte glucose transporter (GLUT1) in the yeast Saccharomyces cerevisiae. Biochem. Soc. Trans..

[202] Schmidl S., Ursu O., Iancu C.V., Oreb M., Oprea T.I., Choe J.Y. (2021). Identification of new GLUT2-selective inhibitors through in silico ligand screening and validation in eukaryotic expression systems. Sci. Rep..

[203] Kasahara T., Maeda M., Boles E., Kasahara M. (2009). Identification of a key residue determining substrate affinity in the human glucose transporter GLUT1. Biochim. Biophys. Acta.

[204] Iancu C.V., Bocci G., Ishtikhar M., Khamrai M., Oreb M., Oprea T.I., Choe J.Y. (2022). GLUT3 inhibitor discovery through in silico ligand screening and in vivo validation in eukaryotic expression systems. Sci. Rep..

[205] Tripp J., Essl C., Iancu C.V., Boles E., Choe J.Y., Oreb M. (2017). Establishing a yeast-based screening system for discovery of human GLUT5 inhibitors and activators. Sci. Rep..

[206] Costa M., Rosell A., Alvarez-Marimon E., Zorzano A., Fotiadis D., Palacin M. (2013). Expression of human heteromeric amino acid transporters in the yeast Pichia pastoris. Protein Expr. Purif..

[207] Kantipudi S., Jeckelmann J.M., Ucurum Z., Bosshart P.D., Fotiadis D. (2020). The Heavy Chain 4F2hc Modulates the Substrate Affinity and Specificity of the Light Chains LAT1 and LAT2. Int. J. Mol. Sci..

[208] Kassem N., Kassem M.M., Pedersen S.F., Pedersen P.A., Kragelund B.B. (2020). Yeast recombinant production of intact human membrane proteins with long intrinsically disordered intracellular regions for structural studies. Biochim. Biophys. Acta Biomembr..

[209] Theis S., Doring F., Daniel H. (2001). Expression of the myc/His-tagged human peptide transporter hPEPT1 in yeast for protein purification and functional analysis. Protein Expr. Purif..

[210] Hu Y., Chen X., Smith D.E. (2012). Species-dependent uptake of glycylsarcosine but not oseltamivir in Pichia pastoris expressing the rat, mouse, and human intestinal peptide transporter PEPT1. Drug Metab. Dispos..

[211] Song F., Hu Y., Jiang H., Smith D.E. (2017). Species Differences in Human and Rodent PEPT2-Mediated Transport of Glycylsarcosine and Cefadroxil in Pichia Pastoris Transformants. Drug Metab. Dispos..

[212] Madeo M., Kovacs A.D., Pearce D.A. (2014). The human synaptic vesicle protein, SV2A, functions as a galactose transporter in Saccharomyces cerevisiae. J. Biol. Chem..

[213] Zhang Y., Tian D., Matsuyama H., Hamazaki T., Shiratsuchi T., Terada N., Hook D.J., Walters M.A., Georg G.I., Hawkinson J.E. (2016). Human Adenine Nucleotide Translocase (ANT) Modulators Identified by High-Throughput Screening of Transgenic Yeast. J. Biomol. Screen..

[214] Rodriguez-Sanchez L., Rial E. (2017). The distinct bioenergetic properties of the human UCP1. Biochimie.

[215] Zackova M., Jezek P. (2002). Reconstitution of novel mitochondrial uncoupling proteins UCP2 and UCP3. Biosci. Rep..

[216] Heidkaemper D., Winkler E., Muller V., Frischmuth K., Liu Q., Caskey T., Klingenberg M. (2000). The bulk of UCP3 expressed in yeast cells is incompetent for a nucleotide regulated H+ transport. FEBS Lett..

[217] Hamazaki T., Leung W.Y., Cain B.D., Ostrov D.A., Thorsness P.E., Terada N. (2011). Functional expression of human adenine nucleotide translocase 4 in Saccharomyces cerevisiae. PLoS ONE.

[218] Damaraju V.L., Mowles D., Yao S., Ng A., Young J.D., Cass C.E., Tong Z. (2012). Role of human nucleoside transporters in the uptake and cytotoxicity of azacitidine and decitabine. Nucleosides Nucleotides Nucleic Acids.

[219] Damaraju V.L., Weber D., Kuzma M., Cass C.E., Sawyer M.B. (2016). Selective Inhibition of Human Equilibrative and Concentrative Nucleoside Transporters by BCR-ABL Kinase Inhibitors: Identification of key hENT1 amino acid residues for interaction with BCR-ABL kinase inhibitors. J. Biol. Chem..

[220] Damaraju S., Zhang J., Visser F., Tackaberry T., Dufour J., Smith K.M., Slugoski M., Ritzel M.W., Baldwin S.A., Young J.D. (2005). Identification and functional characterization of variants in human concentrative nucleoside transporter 3, hCNT3 (SLC28A3), arising from single nucleotide polymorphisms in coding regions of the hCNT3 gene. Pharm. Genom..

[221] SenGupta D.J., Unadkat J.D. (2004). Glycine 154 of the equilibrative nucleoside transporter, hENT1, is important for nucleoside transport and for conferring sensitivity to the inhibitors nitrobenzylthioinosine, dipyridamole, and dilazep. Biochem. Pharmacol..

[222] Vickers M.F., Kumar R., Visser F., Zhang J., Charania J., Raborn R.T., Baldwin S.A., Young J.D., Cass C.E. (2002). Comparison of the interaction of uridine, cytidine, and other pyrimidine nucleoside analogues with recombinant human equilibrative nucleoside transporter 2 (hENT2) produced in Saccharomyces cerevisiae. Biochem. Cell Biol..

[223] Vickers M.F., Mani R.S., Sundaram M., Hogue D.L., Young J.D., Baldwin S.A., Cass C.E. (1999). Functional production and reconstitution of the human equilibrative nucleoside transporter (hENT1) in Saccharomyces cerevisiae. Interaction of inhibitors of nucleoside transport with recombinant hENT1 and a glycosylation-defective derivative (hENT1/N48Q). Biochem. J..

[224] Visser F., Vickers M.F., Ng A.M., Baldwin S.A., Young J.D., Cass C.E. (2002). Mutation of residue 33 of human equilibrative nucleoside transporters 1 and 2 alters sensitivity to inhibition of transport by dilazep and dipyridamole. J. Biol. Chem..

[225] Aoki K., Ishida N., Kawakita M. (2001). Substrate recognition by UDP-galactose and CMP-sialic acid transporters. Different sets of transmembrane helices are utilized for the specific recognition of UDP-galactose and CMP-sialic acid. J. Biol. Chem..

[226] Newstead S., Kim H., von Heijne G., Iwata S., Drew D. (2007). High-throughput fluorescent-based optimization of eukaryotic membrane protein overexpression and purification in Saccharomyces cerevisiae. Proc. Natl. Acad. Sci. USA.

[227] Sun-Wada G.H., Yoshioka S., Ishida N., Kawakita M. (1998). Functional expression of the human UDP-galactose transporters in the yeast Saccharomyces cerevisiae. J. Biochem..

[228] Segawa H., Kawakita M., Ishida N. (2002). Human and Drosophila UDP-galactose transporters transport UDP-N-acetylgalactosamine in addition to UDP-galactose. Eur. J. Biochem..

[229] Ashikov A., Routier F., Fuhlrott J., Helmus Y., Wild M., Gerardy-Schahn R., Bakker H. (2005). The human solute carrier gene SLC35B4 encodes a bifunctional nucleotide sugar transporter with specificity for UDP-xylose and UDP-N-acetylglucosamine. J. Biol. Chem..

[230] Muraoka M., Kawakita M., Ishida N. (2001). Molecular characterization of human UDP-glucuronic acid/UDP-N-acetylgalactosamine transporter, a novel nucleotide sugar transporter with dual substrate specificity. FEBS Lett..

[231] Becares E.R., Pedersen P.A., Gourdon P., Gotfryd K. (2021). Overproduction of Human Zip (SLC39) Zinc Transporters in Saccharomyces cerevisiae for Biophysical Characterization. Cells.

[232] Breen C.J., Martin D.S., Ma H., McQuaid K., O’Kennedy R., Findlay J.B. (2015). Production of functional human vitamin A transporter/RBP receptor (STRA6) for structure determination. PLoS ONE.

[233] King K.M., Damaraju V.L., Vickers M.F., Yao S.Y., Lang T., Tackaberry T.E., Mowles D.A., Ng A.M., Young J.D., Cass C.E. (2006). A comparison of the transportability, and its role in cytotoxicity, of clofarabine, cladribine, and fludarabine by recombinant human nucleoside transporters produced in three model expression systems. Mol. Pharmacol..

[234] Rodriguez L., Batlle A., Di Venosa G., MacRobert A.J., Battah S., Daniel H., Casas A. (2006). Study of the mechanisms of uptake of 5-aminolevulinic acid derivatives by PEPT1 and PEPT2 transporters as a tool to improve photodynamic therapy of tumours. Int. J. Biochem. Cell Biol..

[235] Scalise M., Pochini L., Console L., Losso M.A., Indiveri C. (2018). The Human SLC1A5 (ASCT2) Amino Acid Transporter: From Function to Structure and Role in Cell Biology. Front. Cell Dev. Biol..

[236] Schmidl S., Iancu C.V., Choe J.Y., Oreb M. (2018). Ligand Screening Systems for Human Glucose Transporters as Tools in Drug Discovery. Front. Chem..

[237] Boles E., Oreb M. (2018). A Growth-Based Screening System for Hexose Transporters in Yeast. Methods Mol. Biol..

